# Executive Functioning, Internalizing and Externalizing Symptoms: Understanding Developmental Dynamics Through Panel Network Approaches

**DOI:** 10.1016/j.jaacop.2023.11.001

**Published:** 2023-12-04

**Authors:** René Freichel, Janine Pfirrmann, Peter J. de Jong, Janna Cousijn, Ingmar H.A. Franken, Albertine J. Oldehinkel, Ilya M. Veer, Reinout W. Wiers

**Affiliations:** a University of Amsterdam, Amsterdam, the Netherlands; b University of Groningen, Groningen, the Netherlands; c University Medical Center Groningen, Groningen, the Netherlands; d Erasmus University Rotterdam, Rotterdam, the Netherlands

**Keywords:** executive functioning, externalizing, internalizing, network

## Abstract

**Objective:**

Early adolescence is a transition period during which many mental health disorders emerge. The interplay between different internalizing and externalizing mental health problems in adolescence is poorly understood at the within-person level. Executive functioning (EF) in early adolescence has been shown to constitute a transdiagnostic risk factor, but the specificity of the associations between different domains of EF and mental health problems remains unclear.

**Method:**

Network dynamics (ie, temporal effects) of different internalizing and externalizing symptoms were investigated leveraging data from the Tracking Adolescents’ Individual Lives Survey (TRAILS), a large longitudinal panel study of adolescents (>1,641 participants) assessed at ages 11, 13, and 15. Two novel methodological panel network approaches were used: cross-lagged panel network models and graphical vector autoregressive models. Hierarchical regression models were used to investigate prospective associations between different measures of EF and broadband transdiagnostic dimensions.

**Results:**

Depressive problems predicted a range of other internalizing symptoms (ie, panic, somatic problems, separation anxiety, general anxiety, social phobia) over time, particularly during early adolescence. Important feedback loops with reciprocal associations between different anxiety symptoms were identified. Different facets of EF assessed at age 11, particularly sustained attention, showed weak but significant prospective associations with internalizing and externalizing symptoms at age 13.

**Conclusion:**

The present findings emphasize the importance of targeting depressive problems in early adolescence to prevent a spiral of different internalizing symptoms from arising later on.

Early adolescence is a key transition period during which the prevalence of a range of mental health problems increases and in which top-down executive functions have been proposed to play a role.^1^ Globally, approximately 25% to 31% of adolescents experience a common mental health disorder,^2^ making adolescent psychopathology a substantial burden of disease and a growing source of disability.^3^ The vast majority of mental health disorders diagnosed in adults, including internalizing and externalizing problems, emerge during adolescence and have been associated with a range of negative long-term consequences.^4^ Thus, studying the emergence of different mental health problems during adolescence is crucial to better identify early risk factors and the deleterious cycles that may contribute to persistence of symptoms.

Symptoms of adolescent mental health problems are highly comorbid and have been shown to predict each other over time.^5^ This high level of comorbidity and interdependence between symptoms of different disorders motivated the development of novel methodological frameworks, such as the symptom network approach.^6^ The network approach poses that psychopathology arises from complex interactions between symptoms, both within the same time window and across time. The symptom interactions (edges) between distinct symptom clusters (eg, internalizing–externalizing) are called bridge symptoms and might have an important role in the emergence of comorbidities.^7^ Therefore, panel network models are uniquely equipped to provide mechanistic insights into the development of psychopathology during key transition periods, such as adolescence.

To date, numerous studies have examined cross-sectional associations between internalizing and externalizing symptoms.^8^^,^^9^ Developmental process models, such as the developmental cascade model,^10^ have been proposed to explain symptom dynamics according to which externalizing problems primarily predict subsequent internalizing symptoms, rather than the reverse direction. Adolescent externalizing behaviors, such as delinquency and aggression, may give rise to negative responses from the adolescent’s environment, such as social rejection, academic difficulties, or punishment, which, in turn, may precede internalizing symptoms, such as anxiety or depression.^11^^,^^12^ Countless studies found sex differences in the prevalence of adolescent internalizing (ie, higher prevalence among girls)^13^ and externalizing (ie, higher prevalence among boys) symptoms.^14^ Moreover, there is evidence suggesting sex differences in the developmental trajectories of externalizing problems across adolescence.^15^ Little is known about sex differences in developmental cascades (ie, predictive associations between internalizing and externalizing symptoms). Moreover, most studies testing within-person theories have failed to disentangle within- and between-person effects in the symptom associations over time, and thus little is known about the dynamic changes within the network structure and which symptoms bridge the internalizing and externalizing domains at the different stages of adolescence.

Existing studies on the associations between different internalizing and externalizing symptoms show 3 important limitations that we aim to address in the present study. First, most network analysis studies are cross-sectional studies and consequently lack temporal precedence. For instance, a recent network analysis study showed that generalized anxiety disorder symptoms are most central in largely similar symptom network constellations across different age groups (ie, ages 7.5-14).^9^ However, little is known about how different internalizing and externalizing symptoms predict each other over time—temporal associations that are crucial for informing causal inferences. Second, prior evidence for the co-occurrence of heterogeneous symptoms of different disorders is primarily based on between-person relationships. As widely noted, understanding mechanistic pathways traditionally entails an examination of isolated within-person (change within individuals) effects that form the basis for intervention efforts (within-person change).^16^ Third, existing studies on the co-occurrence and interplay between internalizing and externalizing symptoms are mostly restricted to the later stages of adolescence and early adulthood. Our study is the first to examine contemporaneous (within the same time window) and temporal associations across a 6-year period in early adolescence (ages 11-16).

A core risk factor for the development of internalizing and externalizing mental health problems may be relatively suboptimal executive functioning (EF) in early adolescence. Impairments of EF, including working memory, response inhibition, cognitive flexibility, and sustained attention,^17^ have been associated with elevated internalizing and externalizing symptoms. Moreover, a general psychopathology factor (p factor^18^) has been associated with poorer EF.^19^ However, existing studies primarily examined associations between single executive functions (eg, working memory) and symptoms in isolation. Little is known about the specificity of associations between executive functions and the development of internalizing and externalizing symptoms at different stages of adolescence.

Using a longitudinal panel dataset of more than 2,100 adolescents, our study aimed to examine the dynamic interplay between a range of internalizing and externalizing mental health problems. Based on developmental cascade models, we predicted that externalizing problems should largely predict internalizing problems over time. We used 2 different panel network analytical approaches in parallel. First, cross-lagged panel network (CLPN) models were used to examine age-specific and wave-by-wave changes in the network structure while conflating within- and between-person effects. Second, panel graphical vector autoregressive (GVAR) models were used to separate within- and between-person effects in the overall network structure. The parallel use of both approaches provided us with a unique perspective for understanding developmental cascades at both the intraindividual level (ie, individuals’ within-person deviations from the mean) and the interindividual level (ie, individuals’ scores on a variable relate to scores of others on another variable). Both statistical approaches serve different goals, namely, the understanding of within-person mechanisms (ie, panel GVAR model) as well as the prediction of future outcomes based on the combined within- and between-person effects (ie, CLPN analysis). Finally, we connected this development of 2 broadband transdiagnostic dimensions (internalizing, externalizing) to different domains of EF assessed at age 11.

## Method

### Data Source and Procedure

We used data from the Tracking Adolescents’ Individual Lives Survey (TRAILS) study, a longitudinal cohort study of Dutch (pre-)adolescents assessed every 2 to 3 years from ages 10 to 11 (wave 1) to ages 28 to 30 (wave 7). The study recruited a representative general population sample from urban and rural areas across 5 municipalities in the Netherlands. The study design, procedure, and sample characteristics are described in detail elsewhere.^20^^,^^21^ The present study used data from the first 3 assessment waves (ages 10-16), considering that all relevant outcome measures have been assessed consecutively with the same instrument during these waves. At each wave, a range of self-report questionnaires and clinical interviews were administered. Neuropsychological and cognitive tasks were administered during the first wave only.

### Measures

#### Youth Self-Report

The Youth Self-Report (YSR)^22^ is a commonly used and well-validated measure of behavioral and emotional problems of children 11 to 19 years old. The YSR consists of 112 items that assess internalizing and externalizing mental health problems on a 3-point scale (0 = not true, 1 = somewhat or sometimes true, 2 = very or often true). The YSR includes 2 broadband domains: Internalizing (31 items; Anxious/Depressed, Withdrawn/Depressed, and Somatic Complaints scales) and Externalizing (32 items; Rule-Breaking Behavior and Aggressive Behavior scales) symptoms. Moreover, there are specific *DSM*-oriented scales for Depressive Problems, Anxious Problems, Somatic Problems, Attention Deficit Hyperactivity Problems, Oppositional Defiant Problems, and Conduct Problems. The respective items were averaged to construct continuous scale scores between 0 (no symptoms present) and 2 (all symptoms always or very present). Our analyses focused on all subscales, excluding the anxiety subscale, as anxiety was assessed in more detail using the Revised Child Anxiety and Depression Scale (RCADS).

#### Revised Child Anxiety and Depression Scale

The RCADS^23^ is a self-report measure of 5 anxiety subtypes and depression symptoms. The questionnaire consists of 47 items that are scored on a 4-point Likert scale (0 = never, 1 = sometimes, 2 = often, 3 = always). The RCADS assessment comprises 6 scales (separation anxiety, generalized anxiety, social phobia, panic disorder, obsessive-compulsive disorder [OCD], major depressive disorder) corresponding largely to the *DSM-5* dimensions of anxiety and depressive disorders. The depression scale was not assessed at wave 3. Satisfactory psychometric properties of the RCADS have been well documented, including a replication of the factor structure in the TRAILS sample.^24^

#### Executive Functioning

EF was assessed at ages 10 to 12 (wave 1) using the Amsterdam Neuropsychological Task battery.^25^ The following tasks were included: Sustained Attention Dots task, measuring sustained attention; Shifting Attentional Set–Visual task, measuring response inhibition and cognitive flexibility; and Memory Search Letters task, measuring working memory maintenance. Following previous recommendations on these tasks,^26^ we removed all reaction time (RT) and accuracy measures (outlier) with an absolute *z* score larger than or equal to 4.

#### Sustained Attention Dots

The Sustained Attention Dots task assesses the ability to sustain attention over time.^25^ On each trial, participants are randomly presented with a 3-, 4-, or 5-dot pattern and instructed to press the yes button for a target (4-dot) and the no button for other (3-dot or 5-dot) patterns. An auditory feedback signal is provided for erroneous responses. The task consists of 600 visual dot patterns that are presented across 50 series of 12 trials each. There are 200 trials for each type of stimulus (3-, 4-, or 5-dot pattern), resulting in a 1:2 target-to-nontarget ratio. The primary outcome measure of the task is the within-person standard deviation of the mean RT of 50 series (fluctuation in tempo) as well as the overall percentage of errors. Higher scores on these performance measures indicate worse sustained attention.

#### Shifting Attentional Set–Visual

The Shifting Attentional Set–Visual task measures 2 components of shifting attention: response inhibition and cognitive flexibility.^25^ The task comprises 3 parts in which participants mimic the direction of jumping squares by clicking the left or right mouse button. In part 1, one square is green and jumps randomly (fixed-compatible condition); in part 2, the square is red, and participants must mirror the direction (fixed-incompatible condition); in part 3, the square can be green or red and participants must adapt their response based on the color of the square (random condition). Response inhibition is calculated as the difference in mean RT/percentage of errors between part 2 and part 1.^27^ Cognitive flexibility is computed by subtracting the mean RT/percentage of errors of part 1 from part 3. Higher difference scores indicate weaker cognitive flexibility.

#### Memory Search Letters

The Memory Search Letters task assesses working memory maintenance in varying memory load and distraction.^25^ It consists of 3 parts in which participants memorize 1, 2, or 3 target letters. Participants must decide whether 4 letters on the screen contain the target letter. Target and nontarget trials alternate in random order. Working memory maintenance is computed as the difference between part 3 (3 target letters) and part 1 (1 target letter) mean RT/percentage of error.^27^ Higher difference scores indicate worse working memory maintenance.

### Data Analysis

To examine how different internalizing and externalizing symptoms predict each other over time, we used 2 distinct panel network analytical approaches. First, we implemented a CLPN analysis approach following the study by Zainal and Newman^28^ to examine age-specific developmental changes. Second, we used GVAR models that allowed us to separate within- and between-person effects in the network structure and discern contemporaneous and temporal associations. These 2 approaches offer unique advantages (CLPN: wave-by-wave analysis; panel GVAR: within- and between-person effects separation) in studying the symptom interplay. To examine how EF measures assessed at the first wave relate to the development (waves 2-3) of internalizing and externalizing mental health problems, we used hierarchical regression models.

#### CLPN Models

CLPN models^29^ were used to examine the interrelation between all 10 internalizing and externalizing symptoms during the 3 waves of data. CLPN models examine lag-1 cross-lagged relations between all nodes after incorporating autoregressive effects (ie, a node predicting itself over time). Regularized regressions that included the least absolute shrinkage and selection operator with 10-fold cross-validation were used to calculate autoregressive and cross-lagged estimates between 2 consecutive waves (ie, wave 1 to wave 2; wave 2 to wave 3). The least absolute shrinkage and selection operator regularization in the model implements a penalty procedure (using a tuning parameter γ) through which weak coefficients (ie, edges) are set to zero. This estimation method leads to sparser networks with a lower probability of obtaining false-positive edges in the network. Cross-validation was used to select the optimal γ parameter. Directed cross-lagged edges in the temporal networks represent associations between different nodes across time while controlling for all other nodes in the network. Cross-sectional networks were estimated using the EBICglasso algorithm that uses Bayesian information criterion model selection and least absolute shrinkage and selection operator regularization with a hyperparameter set to 0.5 to remove spurious edges from the network structure.

#### GVAR Model

We used panel GVAR network models^30^ to discern temporal (predictions over time) and contemporaneous (within the same time window) associations in the network structure. The panel GVAR model is structurally similar to a random-intercept cross-lagged panel model^31^^,^^32^ and separates within-person (changes within individuals) and between-person (relation between means) effects. We detrended the data for linear and quadratic effects of time and standardized across waves to ensure stationarity, as is commonly done in panel GVAR approaches that focus on the correlational structure of interest.^33^^,^^34^ First, we fitted a saturated model (with all edges) that was pruned with a step-up model search (α = .05) to remove false positives.^35^ Model fit was evaluated according to standard criteria of good fit as indicated by the root mean square error of approximation, comparative fit index, and Tucker-Lewis index. This novel panel GVAR modeling approach yielding average within-person effects ensures that trait-like (between-person) effects are accounted for in the estimation of contemporaneous and temporal networks.

#### Network Centrality Metrics

For the temporal network, we computed the in-strength (ie, sum incoming edge weights) and out-strength (ie, sum outgoing edge weights) for every node. These commonly used measures capture the degree to which variables exert their influence (out-strength, influence) and are being influenced (in-strength, predictability) by other variables in the network. To examine the extent to which different symptoms act as important bridge symptoms in the contemporaneous networks, we computed measures of bridge centrality using the networktools package.^36^ Bridge centrality describes the sum of edge weights between a node in one community (eg, internalizing symptoms) and all other nodes from a different community (eg, externalizing symptoms).

#### Estimation and Model Stability Analysis

There was a substantial dropout of participants throughout the waves (participation rate: wave 2, 96%; wave 3, 76%). We used full information maximum likelihood (FIML) estimation in the panel GVAR and the regression analyses. FIML is a gold-standard approach^37^ that provides unbiased estimates that are similar to multiple imputation procedures assuming data missing at random. We used a case-dropping bootstrapping analysis to examine the stability of the estimated edge weights and centrality measures. Panel network models were estimated and visualized using the R packages glmnet, psychonetrics, and qgraph.^38^, ^39^, ^40^

#### Hierarchical Regression Models

We used hierarchical regression models to investigate how executive functions (measured at wave 1) affect the development of internalizing and externalizing symptoms at wave 2 and wave 3. We first included the aggregate measures of symptoms (at the previous wave) and sex as predictors, then added all EF measures. We compared the variance explained by the 2 models. For instance, for the prediction of internalizing symptoms at wave 2, the nested 2-step models follow the specification, and the same was done for wave 3:

Model 1: Internalizing Symptoms_wave 2_ = Sex + Internalizing Symptoms_wave 1_ + Externalizing Symptoms_wave 1_

Model 2: Internalizing Symptoms_wave 2_ = Sex + Internalizing Symptoms_wave 1_ + Externalizing Symptoms_wave 1_ + Working memory RT_wave 1_ + Working memory Errors_wave 1_ + Response Inhibition Errors_wave 1_ + Response Inhibition RT_wave 1_ + Fluctuation Tempo_wave 1_ + Fluctuation Errors_wave 1_ + Cognitive Flexibility RT_wave 1_ + Cognitive Flexibility Errors_wave 1_

## Results

At the group level, we observed an increase in the average level of externalizing symptoms and a decrease in the average level of internalizing symptoms throughout early and middle adolescence (see Supplement 1, available online). In particular, there was a trend of increasing prevalence of attention-deficit/hyperactivity disorder (ADHD) and decreasing prevalence of separation anxiety during adolescence. See Tables S1 and S2 (available online) for further details regarding the significant changes and proportion of missingness across waves. Table 1 provides relevant sample characteristics. According to the cutoff scores for the YSR, 23% of participants showed a borderline (subclinical) or clinical score for any internalizing mental health problems at wave 1; 12% of individuals showed a borderline or clinical score for any externalizing mental health problem at wave 1.

**Table 1:** Descriptive Sample Characteristics Information

	Wave 1		Wave 2		Wave 3	
	**n**	**(%)**	**n**	**(%)**	**n**	**(%)**
Participants (female)	2,170	(50.92)	2,074	(51.30)	1,641	(53.26)
	**Mean**	**(SD)**	**Mean**	**(SD)**	**Mean**	**(SD)**
Age (y)	10.61	(0.65)	13.07	(0.61)	15.78	(0.77)
**YSR**						
Internalizing scale score	0.36	(0.24)	0.33	(0.24)	0.31	(0.25)
Externalizing scale score	0.27	(0.20)	0.29	(0.20)	0.31	(0.21)
Depressive Problems	0.29	(0.25)	0.27	(0.26)	0.30	(0.27)
Somatic Problems	0.46	(0.33)	0.32	(0.29)	0.26	(0.28)
Attention Deficit Hyperactivity Problems	0.59	(0.36)	0.67	(0.38)	0.68	(0.38)
Oppositional Defiant Problems	0.45	(0.35)	0.46	(0.35)	0.46	(0.35)
Conduct Problems	0.23	(0.20)	0.23	(0.19)	0.24	(0.20)
**RCADS**						
General anxiety disorder	0.66	(0.45)	0.48	(0.43)	0.52	(0.42)
Social phobia	0.78	(0.43)	0.68	(0.46)	0.73	(0.50)
Separation anxiety	0.37	(0.35)	0.24	(0.29)	0.22	(0.26)
Panic disorder	0.43	(0.36)	0.30	(0.32)	0.28	(0.29)
Obsessive-compulsive disorder	0.60	(0.44)	0.34	(0.35)	0.29	(0.36)

Note: RCADS = Revised Child Anxiety and Depression Scale; YSR = Youth Self-Report.

### Contemporaneous Associations Between Internalizing and Externalizing Symptoms

**Figure 1:**
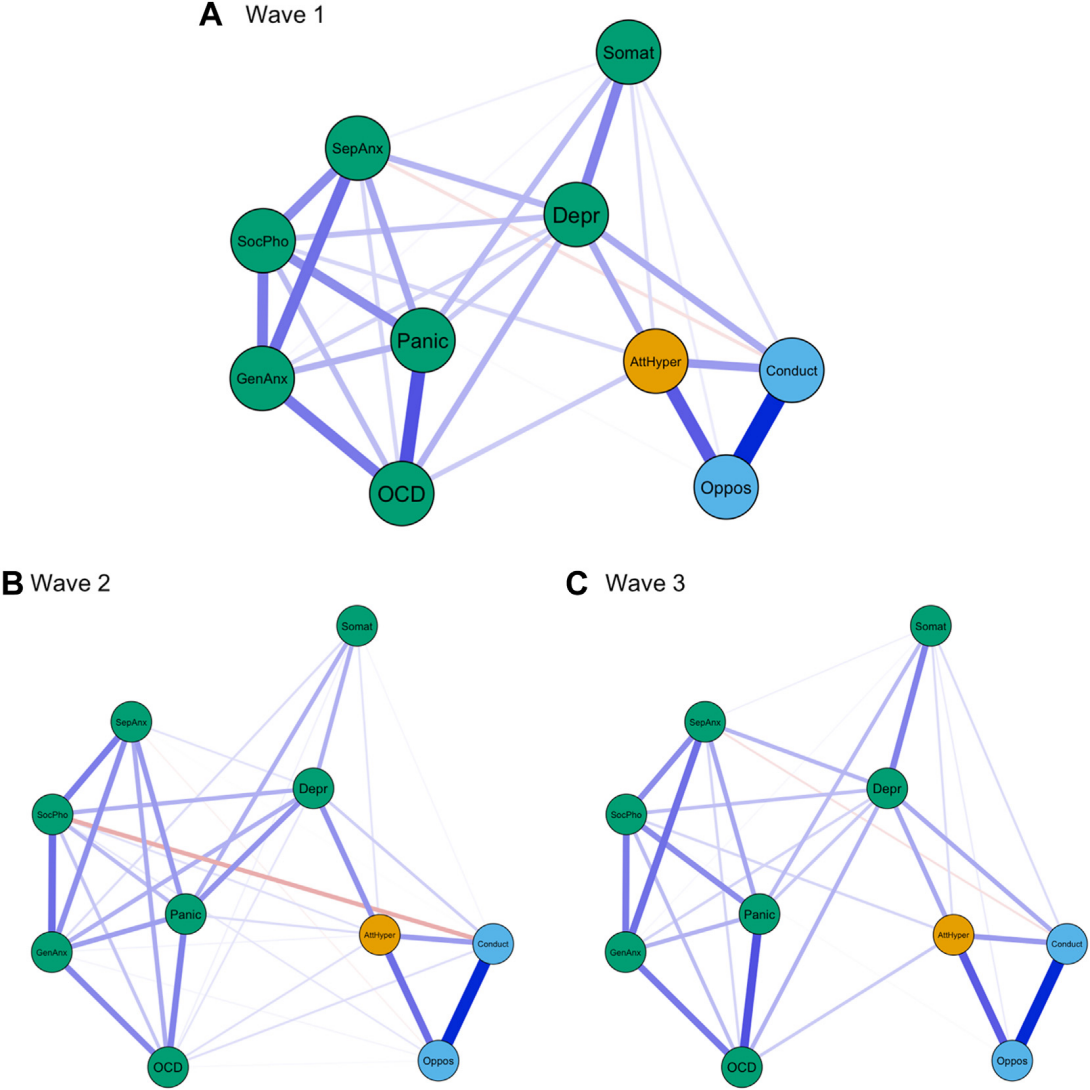
Cross-Sectional Internalizing-Externalizing Networks ***Note:** (A-C) Waves 1-3. Attention problems abbreviations: AttHyper = attention-deficit/hyperactivity disorder; Externalizing abbreviations: Oppos = oppositional defiant problems; conduct = conduct problems; Internalizing abbreviations: Depr = depressive problems; Somat = somatic problems; GenAnx = Generalized Anxiety Disorder; SocPho = social phobia; SepAnx = separation anxiety disorder; Panic = panic disorder; OCD = obessive-compulsive disorder.*

Figure 1 shows cross-sectional associations among internalizing and externalizing symptoms at waves 1, 2, and 3. The overall network structure showed strong similarities across the 3 waves. As expected, internalizing and externalizing symptoms clustered together and showed strong positive associations within their respective cluster. Interestingly, depressive problems emerged as a key bridge symptom connecting a range of internalizing and externalizing symptoms at all 3 waves. Moreover, attention/hyperactivity problems and conduct problems showed moderately strong positive associations with depressive problems and somatic problems. We observed a negative association between social phobia and conduct problems only at wave 2.

To quantify the importance of different bridge symptoms, connecting internalizing and externalizing symptom clusters, we computed a measure of bridge centrality for the contemporaneous networks at all 3 waves (Figure S1, available online). Consistent with the visual inspection, depressive problems constituted the most important internalizing bridge symptom. It should be noted that the importance of depressive problems as bridge symptoms appeared to be decreasing across the three waves.

### Internalizing-Externalizing Symptom Dynamics Over Time

**Figure 2:**
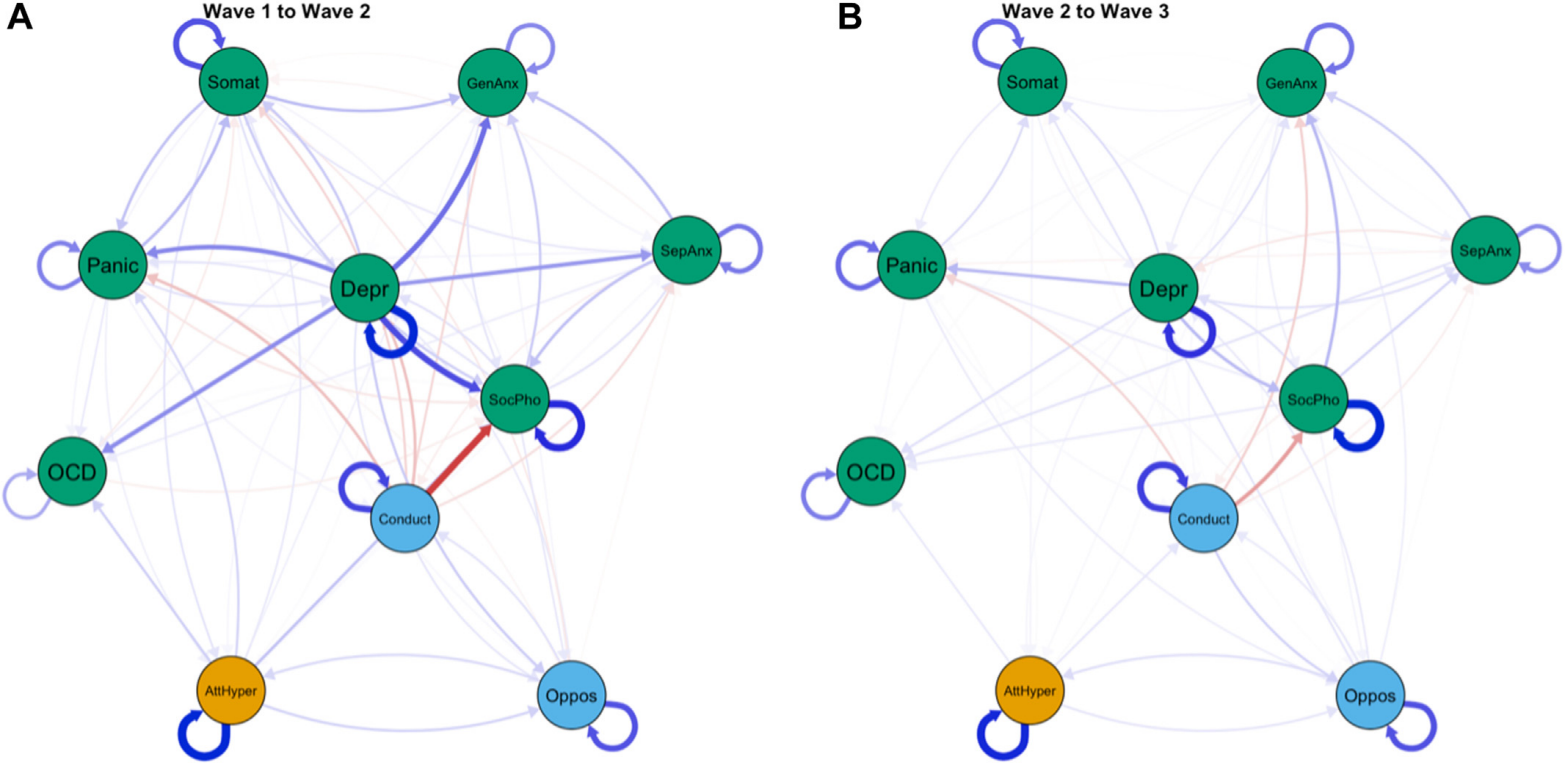
Temporal Internalizing-Externalizing Networks From Cross-Lagged Panel Model ***Note:****(A) Wave 1 to 2. (B, C) Wave 2 to 3. AttHyper = attention deficit hyperactivity; Oppos = oppositional defiant problems; Conduct = conduct problems; Depr = depressive problems; Somat = somatic problems; GenAnx = generalized anxiety disorder; SocPhob = social phobia; SepAnx = separation anxiety disorder; Panic = panic disorder; OCD = obsessive-compulsive disorder.*

We examined temporal associations (Figure 2) separately between waves (ie, wave 1–wave 2; wave 2–wave 3) in the CLPN model. Overall, we observed a complex interplay between different internalizing and externalizing symptoms that predicted each other over time through numerous pathways. During early adolescence (Figure 2A), depressive problems emerged as a key predictor of other internalizing problems (eg, panic disorder, somatic problems, separation anxiety, general anxiety disorder, social phobia, OCD). This influence of depressive problems on other nodes is also shown in the high out-strength (strength of outgoing edges) of depressive problems. The degree of influence of depressive problems on other symptoms was substantially lower during the later stages of adolescence (waves 2-3). Importantly, social phobia symptoms were predicted by a range of other symptoms (eg, lower conduct problems, depressive problems, separation anxiety symptoms). This high level of predictability of social phobia symptoms during later adolescence can also be seen in the high in-strength (strength of incoming edges) (Figures S2, S3, available online). The temporal networks at both change points (wave 1 to wave 2 and wave 2 to wave 3) indicated various reinforcing feedback loops (eg, attention deficit hyperactivity problems–oppositional defiant problems), in which 2 symptoms predict each over time.

**Figure 3:**
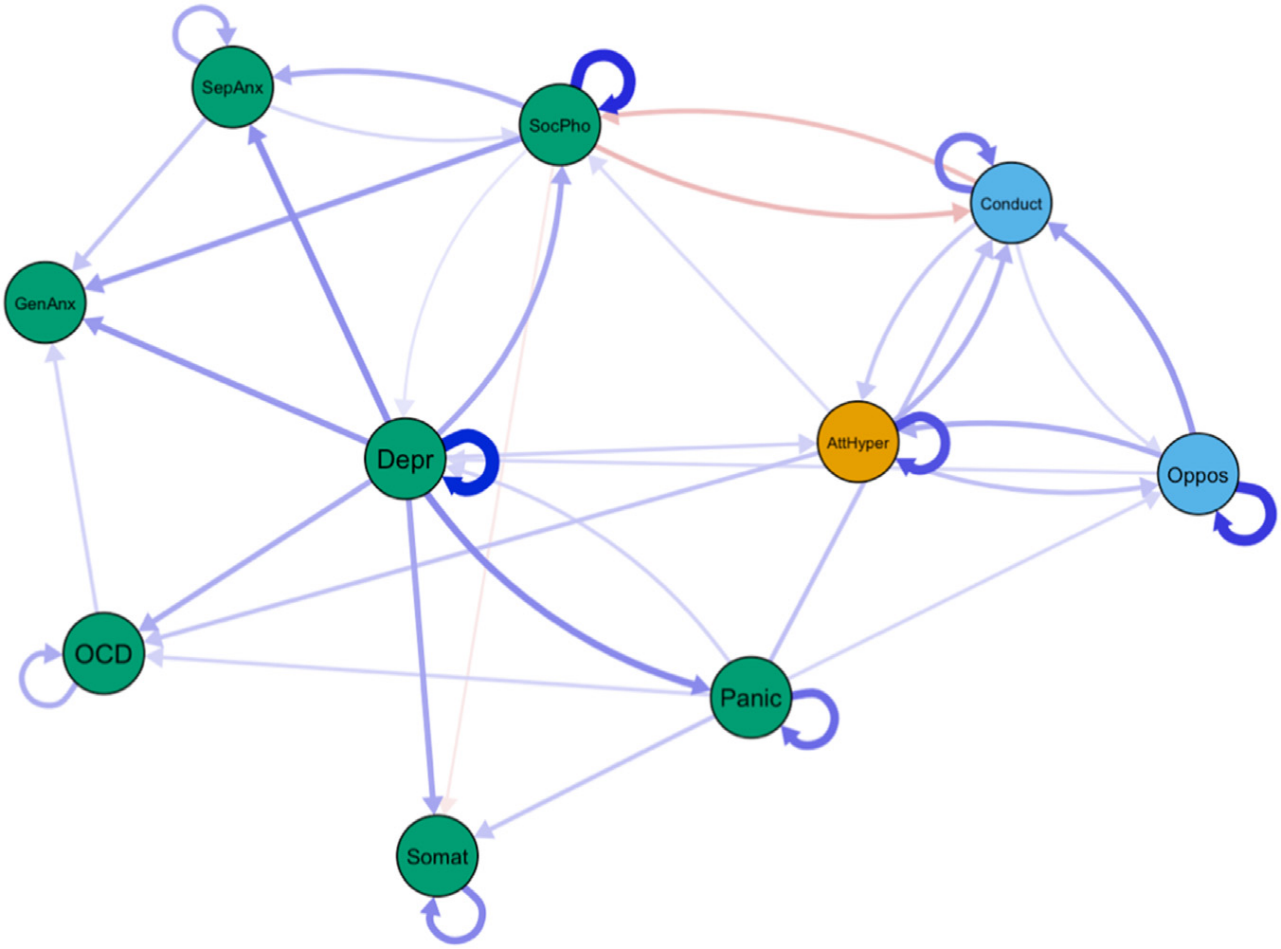
Pruned Temporal Network From Panel Graphical Vector Autoregressive Model ***Note:**AttHyper = attention deficit hyperactivity; Oppos = oppositional defiant problems; Conduct = conduct problems; Depr = depressive problems; Somat = somatic problems; GenAnx = generalized anxiety disorder; SocPhob = social phobia; SepAnx = separation anxiety disorder; Panic = panic disorder; OCD = obsessive-compulsive disorder.*

To validate the findings from our CLPN analyses described above, we used a novel panel GVAR approach that can separate within- and between-person effects in the network structure. The pruned panel GVAR models showed a good fit to the data (root mean square error of approximation = 0.043, comparative fit index = 0.95, Tucker-Lewis index = 0.94). The contemporaneous (Figures S4, S5, available online) and temporal networks (Figure S6, available online) from the panel GVAR analyses generally replicated the central findings from our CLPN analysis at a within-person level. Despite the large similarities, we found some differences between the methods as the directionality of a few associations (eg, conduct problems–social phobia) changed in the panel GVAR model (Figure 3).

The models presented above included 5 different subscales of anxiety assessed through the RCADS measure. As shared method variance may be an issue that could explain the greater within-measure associations found, we conducted sensitivity analyses in which we replaced the 5 RCADS anxiety subscales with the single YSR anxiety subscales (Supplement 2, available online). The results are shown in Figures S7 to S9 (available online). These models largely replicated some of the most important temporal associations (eg, depressive symptoms predicting most other anxiety symptoms) found above.

### Prospective Associations Between EF and Internalizing and Externalizing Symptoms

**Figure 4:**
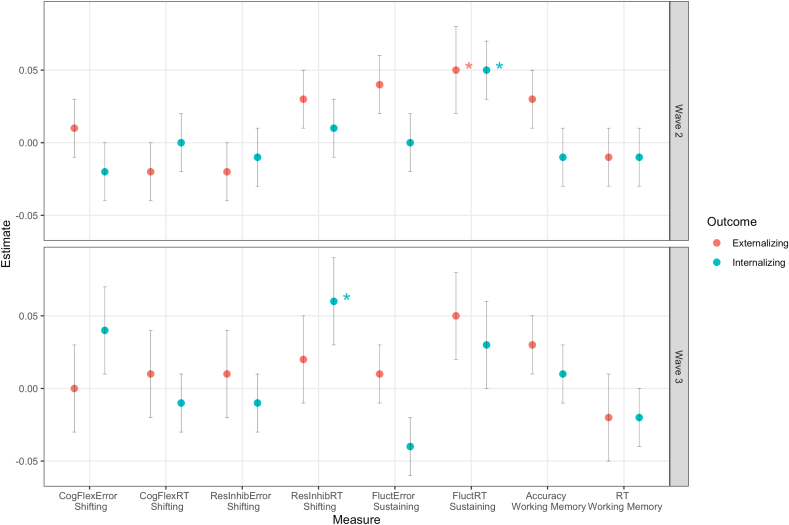
Regression Estimates for Different Executive Functioning Measures ***Note:****The regression estimates refer to the regression models (in step 2) that include sex and previous internalizing and externalizing symptoms as predictors. The standard errors for the regression estimates are shown in vertical bars. CogFlex = cognitive flexibility; Fluct = fluctuations in tempo (sustained attention); ResInhib = response inhibition; RT = reaction times.* *∗*p *< .05*.

Hierarchical regression models were used to estimate associations between specific executive functions (Figure S10, available online) assessed at age wave 1 (mean age 10.61) and the development of internalizing and externalizing symptoms at wave 2 (mean age 13.07) and wave 3 (mean age 15.78). The EF measures were assessed only at wave 1, and thus they could not be integrated into the panel network approaches that require assessments at all respective waves. Overall, the regression models showed significant associations between behavioral measures of sustained attention (speed) at the first wave and future externalizing and internalizing symptoms (see Figure 4 for a visualization of regression coefficients). Higher fluctuations in tempo throughout the Sustained Attention Dots task at wave 1 (indicating worse sustained attention) were associated with higher scores of externalizing and internalizing symptoms at wave 2. Moreover, slower response inhibition/shifting was associated with internalizing symptoms at wave 3, but not at wave 2. All the effect sizes were small (all βs < .07) and the total variance explained in the second-step models that included these EF measures was not substantially larger than the first-step models that included only sex and mental health problems at the previous wave as predictors (Tables S3-S10, available online). No other task measure of attentional shifting (response inhibition accuracy, cognitive flexibility speed, cognitive flexibility accuracy) or working memory was significantly associated with prospective internalizing or externalizing symptoms. Tables S1 through S8 (available online) provide an overview of all regression coefficients.

## Discussion

The present study is the first to our knowledge to investigate the network dynamics of internalizing and externalizing symptoms in early adolescence at the within-person level. Mental health problems showed dynamic patterns of interactions throughout adolescence, in which depressive problems predicted various other internalizing symptoms in particular. Executive functions assessed in early adolescence were differentially associated with the development of internalizing and externalizing symptoms later in adolescence.

Our investigation revealed mostly stable cross-sectional symptom network constellations throughout different stages of adolescence (ages 11-16). Our bootstrapping analysis confirmed sufficient stability of these networks (Figures S11-S13, available online). In line with previous network analysis studies^41^ and the recent Hierarchical Taxonomy of Psychopathology (HiTOP) classification system,^42^ our results support the presence of internalizing and externalizing symptom clusters in which different symptoms of the same domain are strongly co-occurring. Across all 3 waves, we found robust cross-construct associations between ADHD symptoms and anxiety disorder (OCD and social phobia) symptoms (internalizing). This co-occurrence is in line with epidemiological evidence suggesting substantial rates of comorbidity between ADHD and OCD across the life span.^43^ Moreover, we found a negative association between social phobia and conduct problems that is consistent with observations from clinical practice. Individuals with an overwhelming fear of social situations show low levels of aggressive behavior^44^ out of fear of negative judgments by others.

A crucial finding from our results concerns the role of depressive problems as a key bridge symptom (cross-sectional networks) and predictive marker (temporal networks). Depressive problems emerged as the most important bridge symptom, connecting various internalizing symptoms to other externalizing symptoms in the contemporaneous network. The importance of depressive problems as a bridge symptom slightly decreased over the course of adolescence. This finding aligns with reports showing that early-onset depression is associated with high rates of comorbidity with other mental disorders, including anxiety disorders in adolescents.^45^

The temporal networks emphasize the influential role of depressive problems in predicting a range of other internalizing symptoms in early adolescence. This is consistent with the high centrality observed in the contemporaneous network. During the change from age 10 to age 13 (wave 1 to wave 2) in particular, depressive problems predicted higher levels of social phobia, OCD, general anxiety, separation anxiety, panic symptoms, and somatic complaints. Our results are consistent with a recent CLPN analysis indicating depressed mood as an important influencer of other symptoms.^8^ However, we replicate and extend this finding by separating within- and between-person effects that are conflated in CLPN models.^29^ Likely, different cognitive-behavioral processes prevalent in depression may explain this catalyst role of depressive symptoms on other symptom states. For instance, rumination as a core symptom of depression has been shown to predict anxiety symptoms and characterize comorbid depression/anxiety.^46^ Moreover, prior diagnosis of major depressive disorder and negative affect predicted the onset of panic attacks in high school students.^47^ Particularly in early adolescence, low energy, avoidance, and social isolation triggered by depression may lead to the emergence of various anxiety problems that persist throughout adolescence and adulthood. Thus, our findings suggest that targeting adolescent depressive symptoms in clinical practice may prove viable in preventing various other internalizing symptoms arising later in life. To the best of our knowledge, this is one of the first studies showing this catalyst effect of depression on a range of outcomes at the within-person level in early and middle adolescence.

In addition to the prominent role of depressive problems, we identified important feedback loops (reciprocal associations) between key symptoms. For instance, panic symptoms and somatic problems predicted each other over time in both panel network models. The increased body vigilance common in panic attacks may exacerbate negative or threatening interpretations of physical sensations that manifest in somatic problems. This in turn may lead to increased anxiety sensitivity, which constitutes a major risk factor for panic disorder.^48^ Interestingly, we also observed reciprocal associations (in both methods) between ADHD symptoms and oppositional defiant problems that are consistent with bidirectional associations reported in prior literature.^49^ These findings further complement discussions regarding the developmental precursor model of ADHD symptoms^50^ according to which ADHD symptoms predict argumentative/defiant symptoms.

We complemented our analysis of symptom network dynamics during adolescence by examining how EF assessed at age 10 to 11 plays into the development of broadband transdiagnostic dimensions of internalizing and externalizing symptoms. Our results suggest that primarily sustained attention, and no other executive functions such as working memory or attentional shifting, might represent a transdiagnostic risk factor for the development of internalizing and externalizing mental health problems. Our findings extend prior studies that showed cross-sectional or domain-specific (ie, affective problems) associations with sustained attention.^51^^,^^52^ The ability to sustain attention to relevant stimuli or information despite distractions constitutes a core executive function that plays a major role in daily functioning of individuals. Impairments in sustained attention have been associated with a range of mental disorders, including internalizing conditions such as depression^53^ and externalizing conditions such as ADHD.^54^ Our results suggest that sustained attention is an important executive function in early adolescence with negative clinical transdiagnostic repercussions. Shifting attention was associated with more internalizing symptoms only at wave 3. This is consistent with previous links between difficulties shifting attention, response inhibition, and psychopathological processes, such as rumination.^55^ Importantly, we found that the executive functions, including sustained attention, explained no additional variance over and beyond internalizing and externalizing mental health problems at the previous wave. Likely, associations between sustained attention and internalizing/externalizing mental health problems at the previous waves already accounted for these effects. Other executive functions, such as attentional inhibition and working memory, showed no significant associations with the symptom measures. It is possible that these executive functions are associated with mental health problems at other time lags (ie, later adolescence) or show solely disorder-specific associations. Future studies should also consider integrating a common EF factor at each time point into symptom panel networks—a crucial step toward understanding the dynamic interplay that was not feasible in our study considering that EF was assessed only at the first assessment wave. For instance, integrating cognitive measures of sustained attention in the dynamic network models may bridge our understanding of transdiagnostic risk factors as well as transdiagnostic network dynamics.

Our findings should be interpreted in light of several limitations. First, our analyses relied on self-report of adolescents, which may naturally be biased. Moreover, to foster model estimation and identification, we used scale scores that do not account for measurement error. Future studies should include both parent and teacher reports to validate these findings and model all multiple-indicator constructs as latent variables. Second, the 2 methodological panel network approaches are limited by several methodological constraints. A common concern in all network modeling approaches is the assumption of multivariate normality. Restricted variance in some nodes (eg, OCD at wave 3) may affect the estimation of temporal effects.^30^ The CLPN model cannot separate within- and between-person effects, which limits the interpretation of directed temporal effects as mechanistic pathways. The panel GVAR approach overcomes this limitation because it separates within- and between-person effects in the network structure. The resulting temporal associations are Granger causal and fulfill the criteria of temporal precedence; however, these associations might not necessarily indicate causal effects.^56^ Moreover, the panel GVAR modeling approach assumes lag-1 linear dynamics between all variables and an approximately stationary time series, and thus it cannot capture (nonlinear) processes that operate on shorter or broader time scales. Further methodological developments, including methods for formal model comparisons, are needed to better understand the differences in results (eg, the role of oppositional problems) obtained from CLPN and panel GVAR models, which likely concern the separation of within- and between-person effect. Lastly, future studies should adopt confirmatory network modeling strategies to directly replicate and test the predictions emerging from developmental cascade models. We have used FIML estimation to account for missingness in the GVAR and regression models. Although FIML is widely considered an appropriate tool that can produce unbiased parameter estimates,^57^ it cannot overcome the assumption of missing completely at random, and some evidence suggests that attrition in the TRAILS sample is associated with baseline levels of psychopathology.^20^^,^^58^

Our study is the first to our knowledge to investigate the within-person network dynamics between a range of internalizing and externalizing symptoms throughout early adolescence. We showcase the use of 2 novel methodological panel network approaches in parallel, CLPN and panel GVAR models, that are uniquely equipped to explore dynamic patterns of interaction in longitudinal datasets while controlling for many variables. Adolescence remains a consequential period of sensitivity in which different internalizing and externalizing problems emerge and contribute to symptom persistence in adulthood. Our findings identified sustained attention as a transdiagnostic risk factor, and we pinpointed key catalyst symptoms (eg, depressive problems) in early adolescence. Future empirical investigations of these target points in intervention studies may potentially lead to effective intervention efforts that may prevent a deleterious cycle of symptom enhancement from arising.
