# Supplementary material for: Executive Functioning, Internalizing and Externalizing Symptoms: Understanding Developmental Dynamics Through Panel Network Approaches
Source: JAACAP Open. 2023 Dec 4;2(1):66–77. doi: 10.1016/j.jaacop.2023.11.001 (PMC11562421; doi:10.1016/j.jaacop.2023.11.001)
Supplement: Supplement 1 and Supplement 2 [file mmc1.docx]

**Executive Functioning, Internalizing and Externalizing Symptoms – Understanding Developmental Dynamics Through Panel Network Approaches**

**Supplementary Materials**

[Section 1: Trends Across Time 2](#_Toc141782861)

[Section 2: Missingness analysis 3](#_Toc141782862)

[Section 3: Cross-Lagged Panel Network Analysis 4](#_Toc141782863)

[Section 4: Panel GVAR Network Analysis 7](#_Toc141782864)

[Section 5: Sensitivity Analysis (Including YSR Anxiety Subscale) 10](#_Toc141782865)

[Section 6: Executive functioning task regression models 14](#_Toc141782866)

[Section 7: Bootstrapping stability analysis 19](#_Toc141782867)

# Section 1: Trends Across Time

# Supplement 1: To examine how symptoms change across time, we used linear mixed effects models. For every symptom measure, we estimated a separate model that accounted for the repeated measurements within individuals. Table S1 reports the main effects of time compared with the measurement during the first wave.

**Table S1**

*Estimates from mixed effects models to test change across waves*

| **Variable** | **Int (Est)** | **Wave 2 (Est, SE)** | **Wave 3 (Est, SE)** |
| --- | --- | --- | --- |
| Obsessive-compulsive disorder (RCADS) | 0.596 | -0.256 (0.010)*** | -0.308 (0.011)*** |
| Panic disorder (RCADS) | 0.426 | -0.126 (0.008)*** | -0.149 (0.009)*** |
| Separation anxiety (RCADS) | 0.374 | -0.139 (0.007)*** | -0.159 (0.008)*** |
| Social phobia (RCADS) | 0.777 | -0.094 (0.010)*** | -0.063 (0.011)*** |
| General anxiety disorder (RCADS) | 0.587 | 0.083 (0.008)*** | 0.093 (0.009)*** |
| Depressive Problems (YSR) | 0.291 | -0.018 (0.006)* | -0.001 (0.006) |
| Attention deficit hyperactivity (YSR) | 0.59 | 0.083 (0.008)*** | 0.093 (0.009)*** |
| Conduct Problems (YSR) | 0.235 | -0.006 (0.005) | 0.004 (0.005) |
| Oppositional-defiant Problems (YSR) | 0.445 | 0.013 (0.009)*** | 0.016 (0.009)*** |
| Somatic Problems (YSR) | 0.457 | -0.142 (0.008)*** | -0.200 (0.008)*** |
| Externalizing scale score (YSR) | 0.271 | 0.016 (0.005)** | 0.045 (0.005)*** |
| Internalizing scale score (YSR) | 0.363 | -0.036 (0.005)*** | -0.054 (0.006)*** |

*Note*. *** indicates p < 0.001, ** indicates p < 0.01, * indicates p < 0.05. The standard errors (SE) are given in parentheses next to the estimates (EST) for wave 2 (TF2) and wave 3 (TF3). *INT =* intercept. The model examines change in symptoms relative to wave 1. YSR = Youth Self-Report; RCADS = Revised Child Anxiety and Depression Scale.

# Section 2: Missingness analysis

**Table S2**

*Percentage of Missing Values for Each Measure At All Waves*

| **Measure** | **Wave 1** | **Wave 2** | **Wave 3** |
| --- | --- | --- | --- |
| Fluctuations RT (Sustaining) | 0.81 | NA | NA |
| Fluctuations error (Sustaining) | 0.63 | NA | NA |
| Cognitive flexibility RT (Shifting) | 1.03 | NA | NA |
| Response inhibition RT (Shifting) | 0.85 | NA | NA |
| RT working memory | 0.76 | NA | NA |
| Cognitive flexibility error (Shifting) | 1.7 | NA | NA |
| Response inhibition errror (Shifting) | 0.9 | NA | NA |
| Accuracy working memory | 0.67 | NA | NA |
| Internalizing scale score (YSR) | 2.65 | 6.95 | 26.38 |
| Externalizing scale score (YSR) | 1.88 | 6.19 | 25.53 |
| Depressive Problems (YSR) | 1.75 | 6.19 | 25.53 |
| Somatic Problems (YSR) | 2.65 | 7.00 | 26.33 |
| Attention deficit Hyperactivity (YSR) | 1.62 | 6.19 | 25.66 |
| Oppositional Defiant Problems (YSR) | 2.11 | 6.33 | 25.75 |
| Conduct Problems (YSR) | 1.79 | 6.19 | 25.53 |
| General Anxiety (RCADS) | 0.99 | 6.55 | 25.57 |
| Social Phobia (RCADS) | 0.94 | 6.55 | 25.66 |
| Separation Anxiety (RCADS) | 0.94 | 6.55 | 25.66 |
| Panic Disorder (RCADS) | 0.99 | 6.55 | 25.57 |
| Obsessive-compulsive Disorder (RCADS) | 1.08 | 6.68 | 25.66 |

*Note.* RT = Reaction Time. The cognitive measures have only been assessed during the first wave (NA refers to non-applicable). YSR = Youth Self-Report; RCADS = Revised Child Anxiety and Depression Scale.

# Section 3: Cross-Lagged Panel Network Analysis

**Figure S1**

*Bridge Centrality Measure for Contemporaneous Networks*


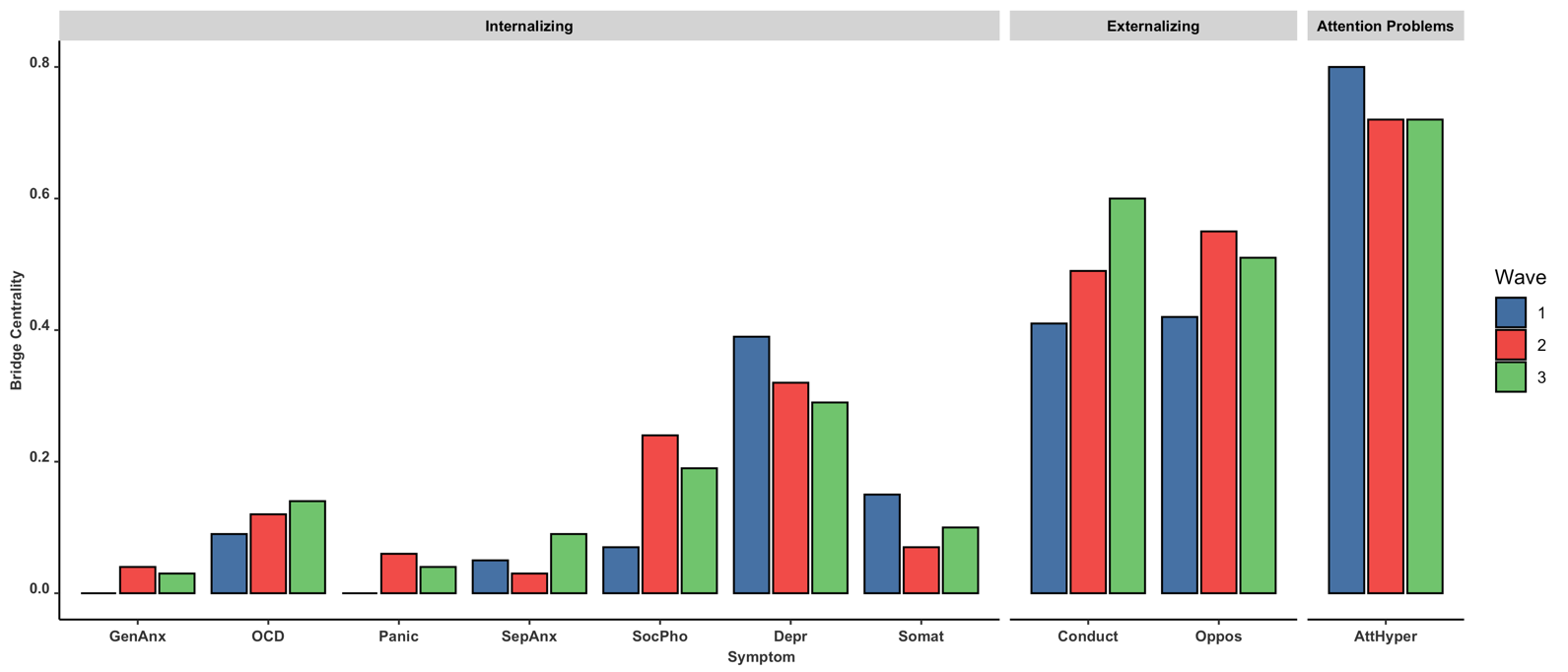


*Note.* GenAnx = General Anxiety, OCD = Obsessive-compulsive Disorder, Panic = Panic Disorder, SepAnx = Separation Anxiety, SocPho = Social Phobia, AttHyper = Attention Deficit Hyperactivity Problems, Depr = Depressive Problems, Conduct = Conduct Problems, Oppos = Oppositional Defiant Problems, Somat = Somatic Problems.

**Figure S2**

*In- and Out-strength Centrality for Temporal Network From Wave 1 to Wave 2 (CLPN Model)*


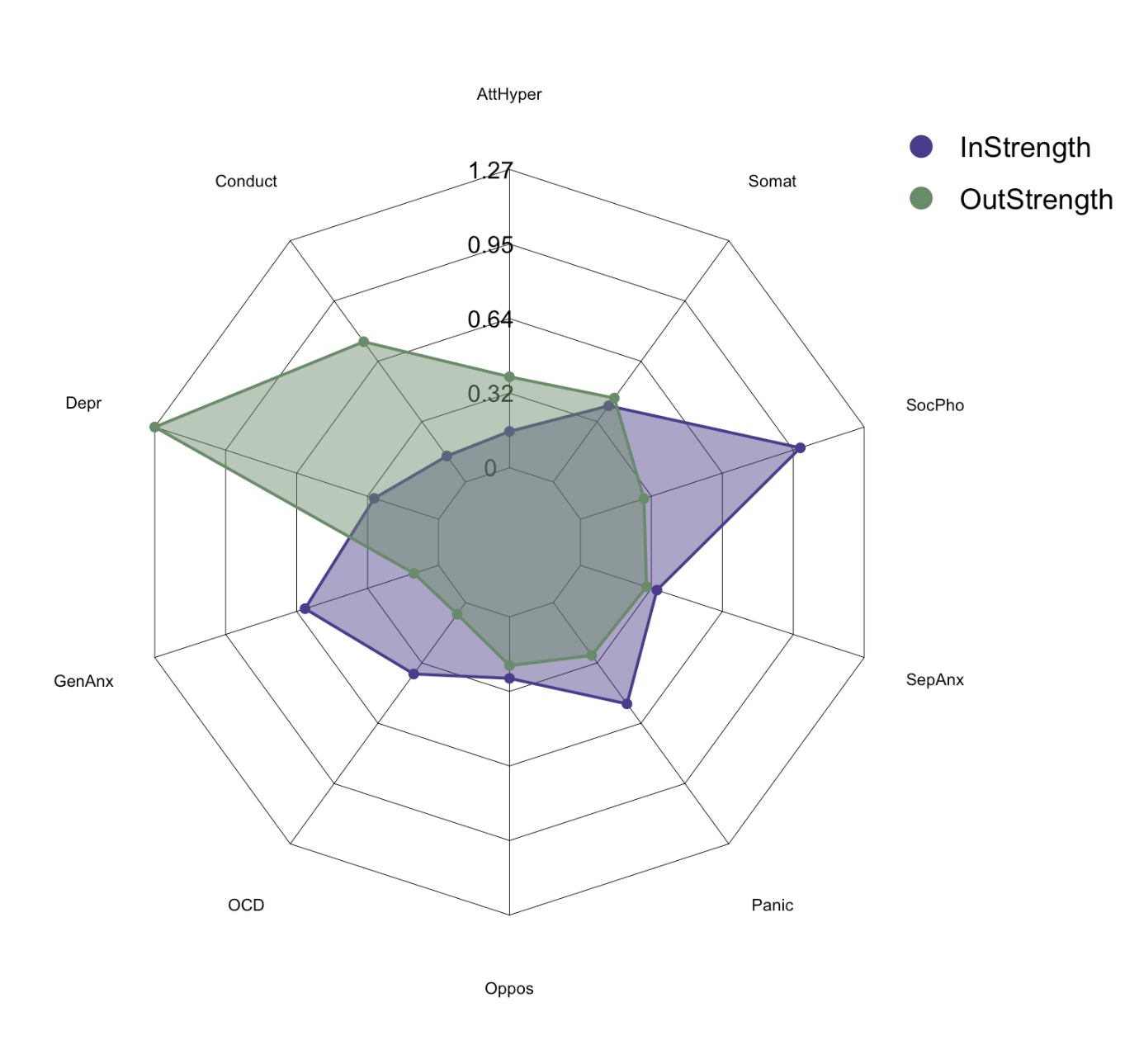


*Note.* GenAnx = General Anxiety, OCD = Obsessive-compulsive Disorder, Panic = Panic Disorder, SepAnx = Separation Anxiety, SocPho = Social Phobia, AttHyper = Attention Deficit Hyperactivity Problems, Depr = Depressive Problems, Conduct = Conduct Problems, Oppos = Oppositional Defiant Problems, Somat = Somatic Problems, CLPN = Cross-Lagged Panel Network Analysis.

**Figure S3**

*In- and Out-strength Centrality for Temporal Network From Wave 2 to Wave 3 (CLPN Model)*


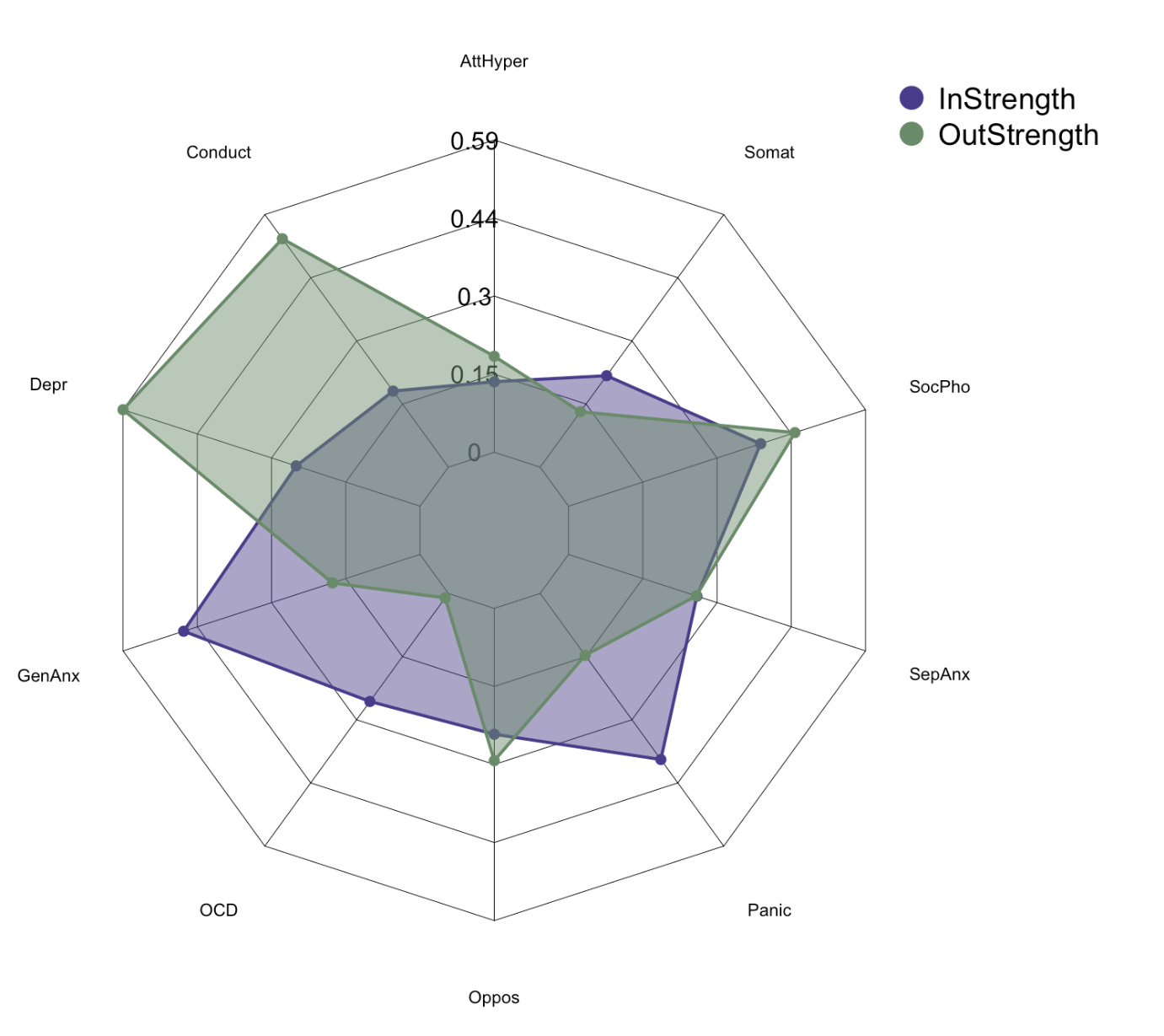


*Note.* GenAnx = General Anxiety, OCD = Obsessive-compulsive Disorder, Panic = Panic Disorder, SepAnx = Separation Anxiety, SocPho = Social Phobia, AttHyper = Attention Deficit Hyperactivity Problems, Depr = Depressive Problems, Conduct = Conduct Problems, Oppos = Oppositional Defiant Problems, Somat = Somatic Problems, CLPN = Cross-Lagged Panel Network Analysis.

# Section 4: Panel GVAR Network Analysis

**Figure S4**

*Pruned Contemporaneous Network (Panel GVAR Model)*

**
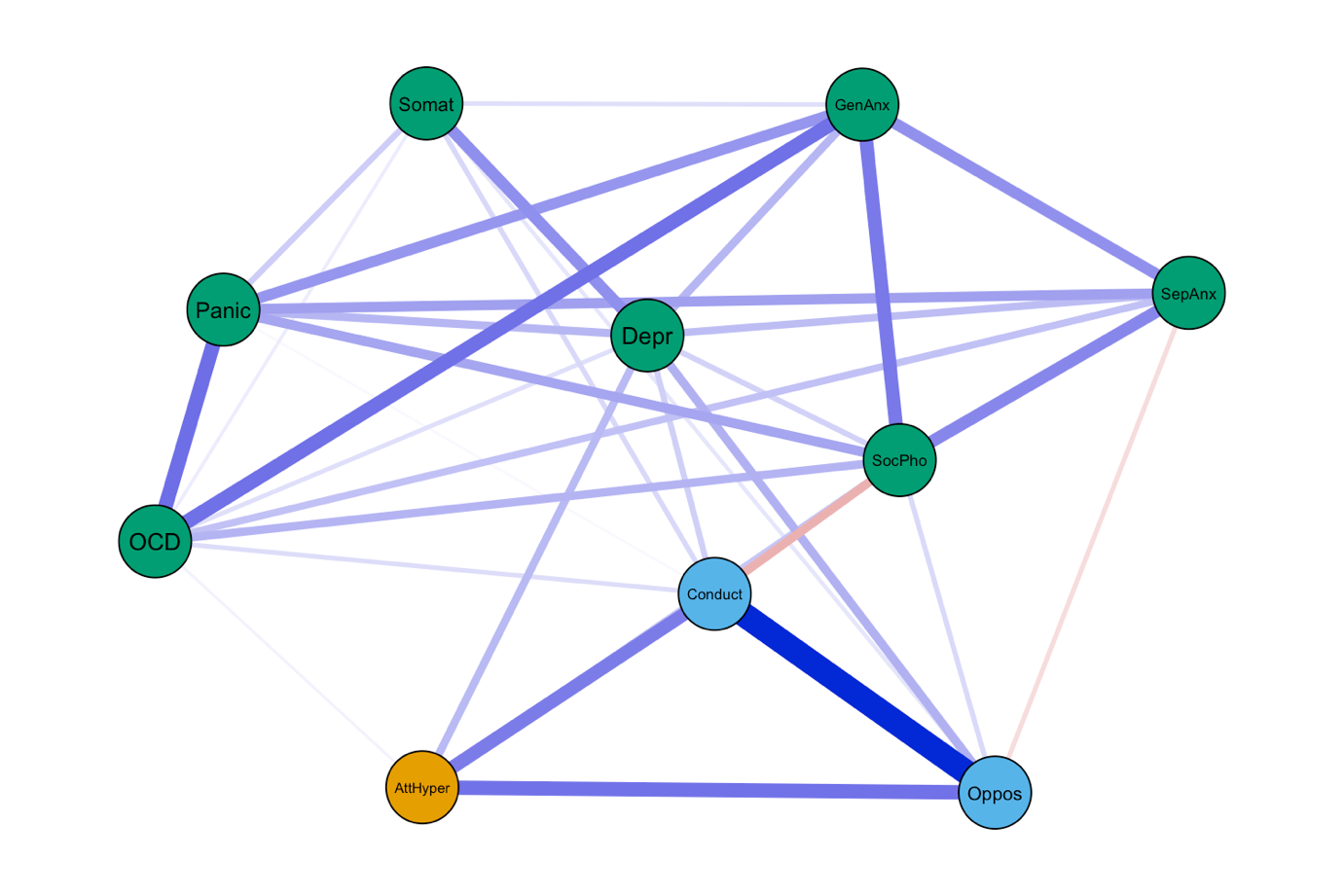
**

*Note.* GenAnx = General Anxiety, OCD = Obsessive-compulsive Disorder, Panic = Panic Disorder, SepAnx = Separation Anxiety, SocPho = Social Phobia, AttHyper = Attention Deficit Hyperactivity Problems, Depr = Depressive Problems, Conduct = Conduct Problems, Oppos = Oppositional Defiant Problems, Somat = Somatic Problems, Panel GVAR = panel Graphical Vector-Autoregression Model.

**Figure S5**

*Bridge Centrality for Pruned Contemporaneous Network (Panel GVAR Model)*

**
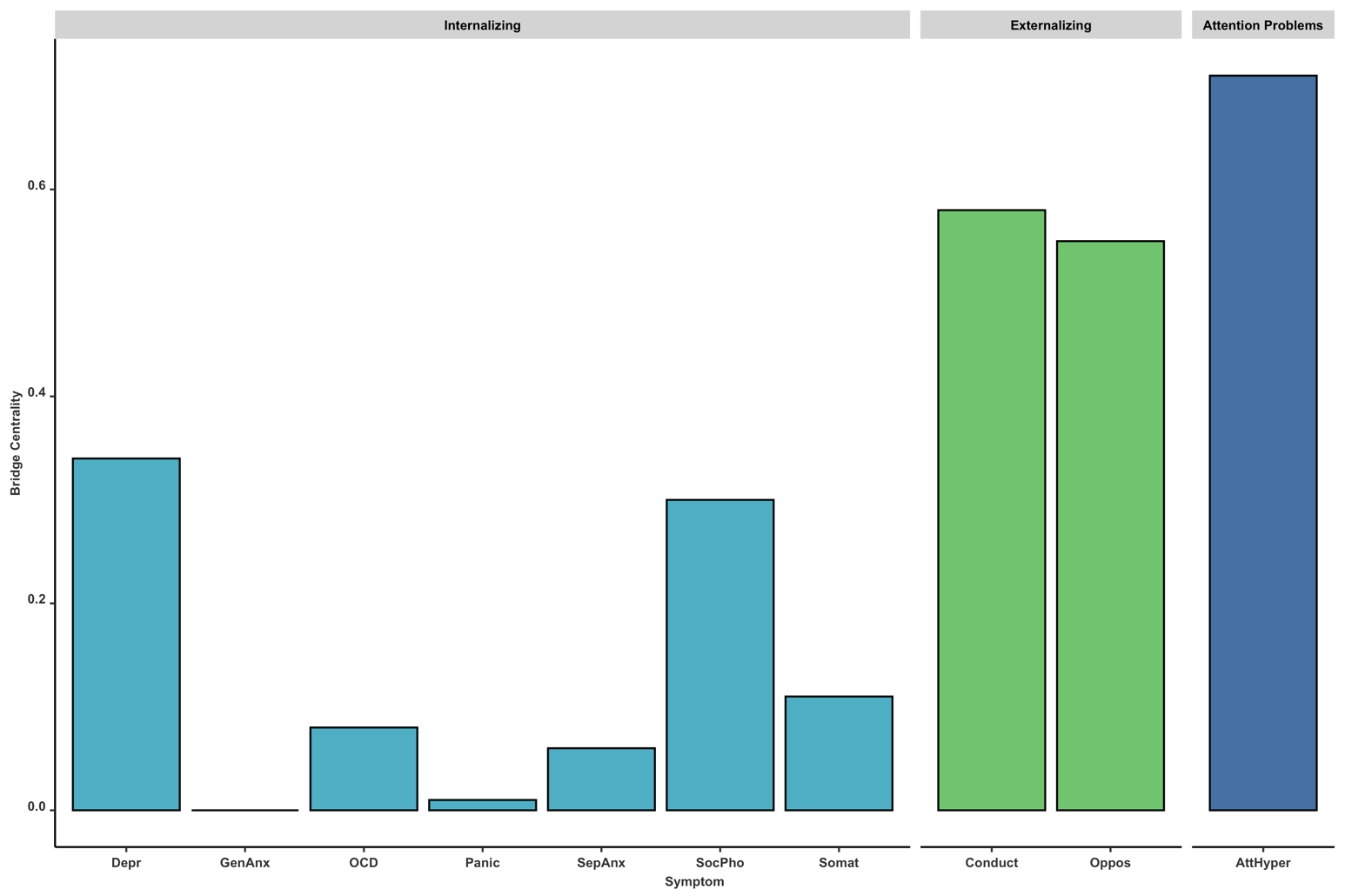
**

*Note.* GenAnx = General Anxiety, OCD = Obsessive-compulsive Disorder, Panic = Panic Disorder, SepAnx = Separation Anxiety, SocPho = Social Phobia, AttHyper = Attention Deficit Hyperactivity Problems, Depr = Depressive Problems, Conduct = Conduct Problems, Oppos = Oppositional Defiant Problems, Somat = Somatic Problems, Panel GVAR = panel Graphical Vector-Autoregression Model.

**Figure S6**

*In- and Out-Strength Centrality for Pruned Temporal Network (Panel GVAR Model)*

*
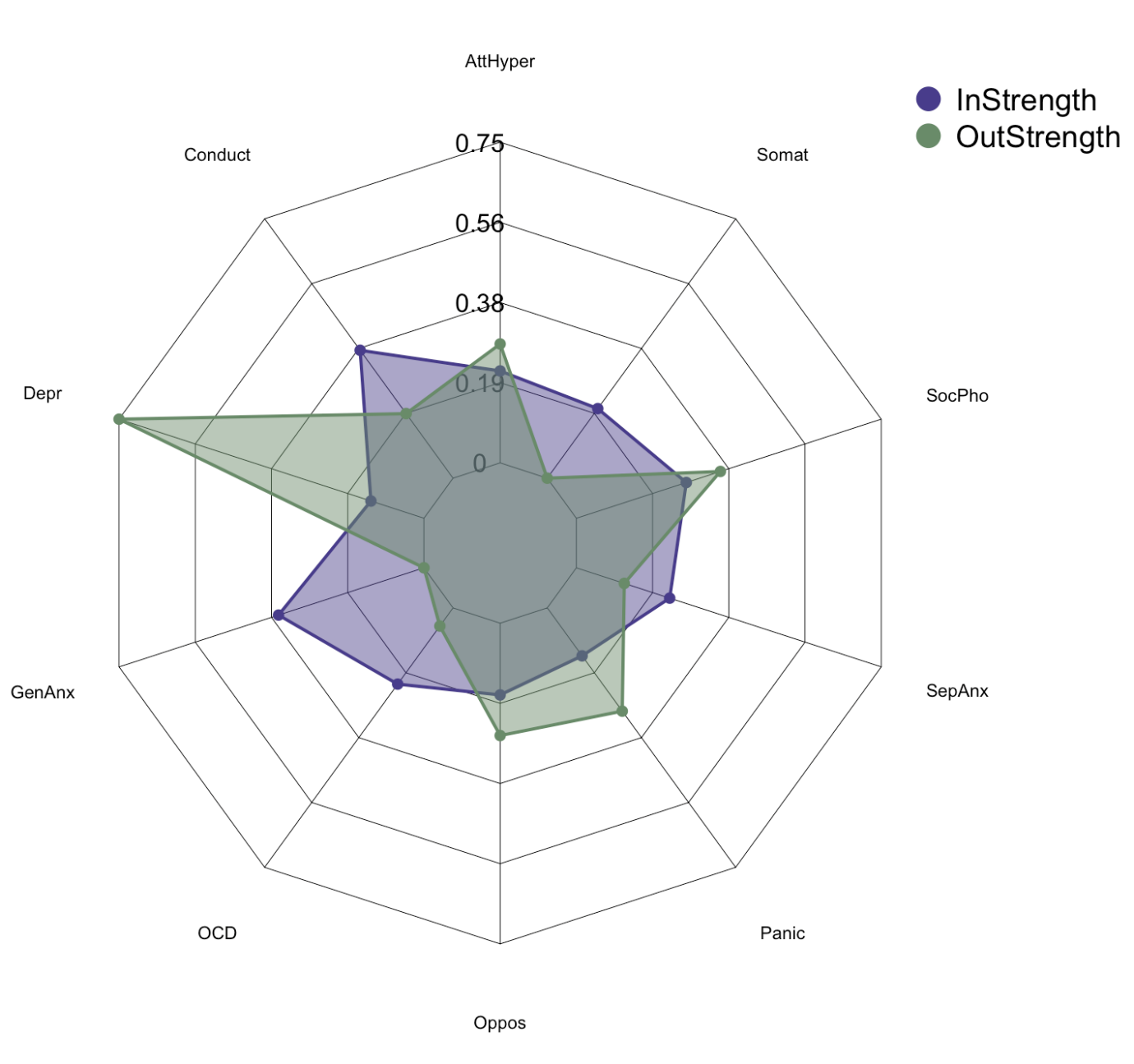
*

*Note.* GenAnx = General Anxiety, OCD = Obsessive-compulsive Disorder, Panic = Panic Disorder, SepAnx = Separation Anxiety, SocPho = Social Phobia, AttHyper = Attention Deficit Hyperactivity Problems, Depr = Depressive Problems, Conduct = Conduct Problems, Oppos = Oppositional Defiant Problems, Somat = Somatic Problems, Panel GVAR = panel Graphical Vector-Autoregression Model.

# Section 5: Sensitivity Analysis (Including YSR Anxiety Subscale)

**Supplement 2:** The models presented in the main text contain the five separate RCADS anxiety subscales. While this approach allows us to parse heterogeneity underlying different anxiety disorders, there may be shared method variance that could explain the greater within-measure associations found. To address this concern, we repeated the same set of analyses using the YSR anxiety subscale instead of the five separate RCADS anxiety subscales. The model and analysis specifications are the same as the ones presented in the manuscript:

**Panel GVAR model.**

The panel GVAR model showed excellent fit to the data (RMSEA = 0.042, CFI = 0.96, TLI = 0.96). The temporal and contemporaneous networks are shown in Figures S7 and S8 respectively.

**Figure S7**

*Pruned Contemporaneous Network (Panel GVAR Model) With YSR Anxiety Subscale
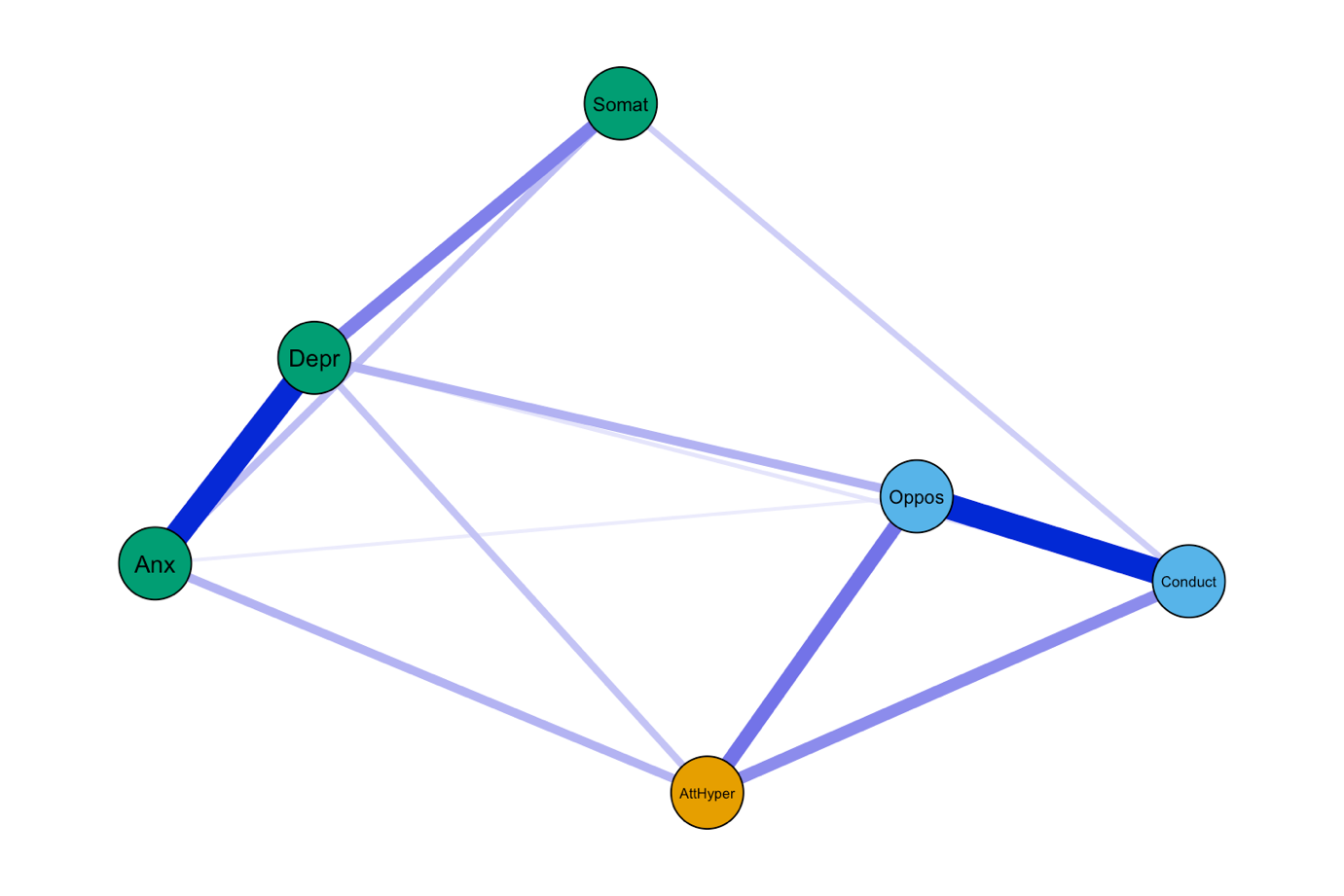
Note.* GenAnx = General Anxiety, OCD = Obsessive-compulsive Disorder, Panic = Panic Disorder, SepAnx = Separation Anxiety, SocPho = Social Phobia, AttHyper = Attention Deficit Hyperactivity Problems, Depr = Depressive Problems, Conduct = Conduct Problems, Oppos = Oppositional Defiant Problems, Somat = Somatic Problems, Panel GVAR = panel Graphical Vector-Autoregression Model.

**Figure S8**

*Pruned Temporal Network (Panel GVAR Model) With YSR Anxiety Subscale*


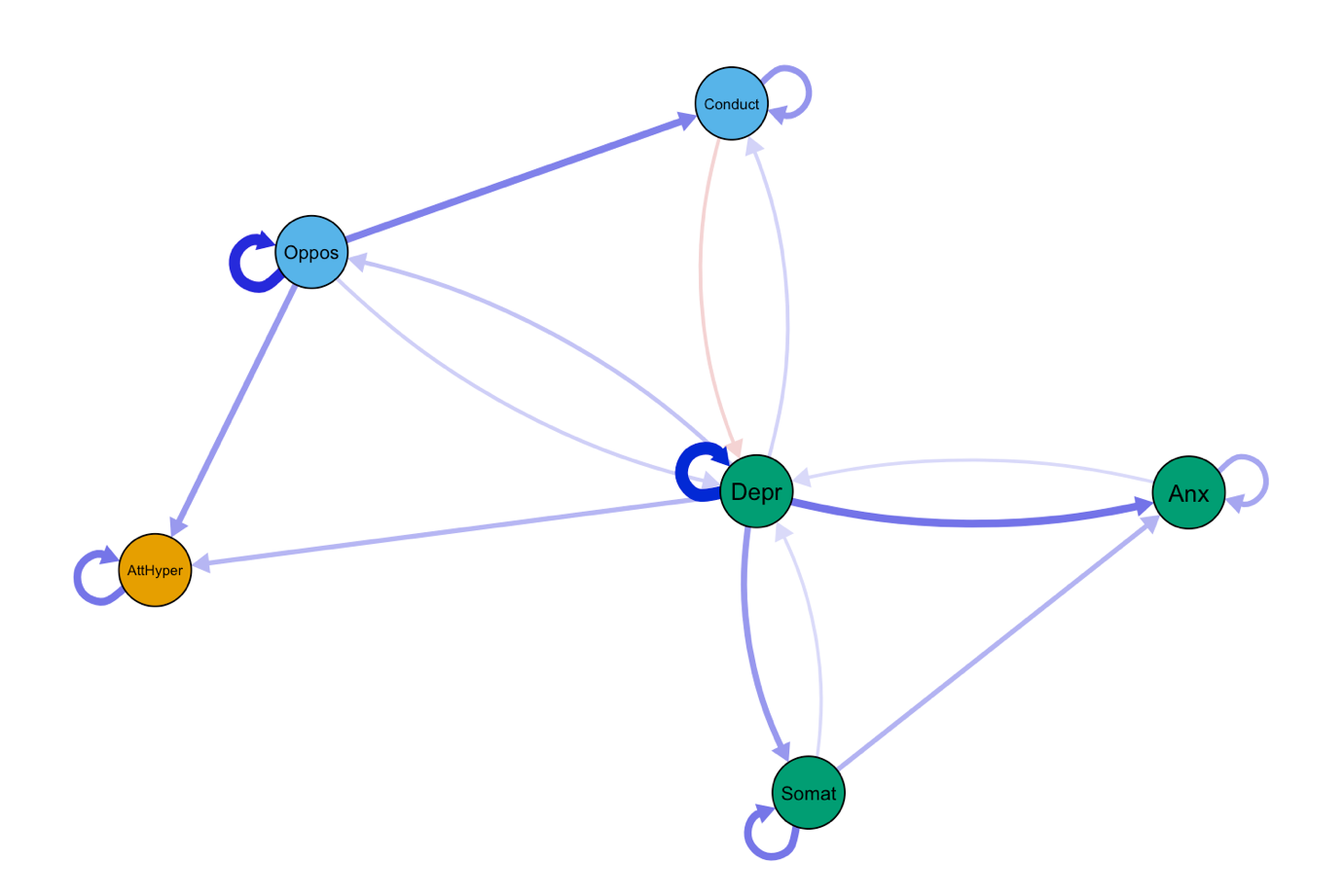


*Note.* GenAnx = General Anxiety, OCD = Obsessive-compulsive Disorder, Panic = Panic Disorder, SepAnx = Separation Anxiety, SocPho = Social Phobia, AttHyper = Attention Deficit Hyperactivity Problems, Depr = Depressive Problems, Conduct = Conduct Problems, Oppos = Oppositional Defiant Problems, Somat = Somatic Problems, Panel GVAR = panel Graphical Vector-Autoregression Model.

**Cross-lagged network analysis.**

**Figure S9**

*Temporal Networks (CLPN Model) With YSR Anxiety Subscale*

**
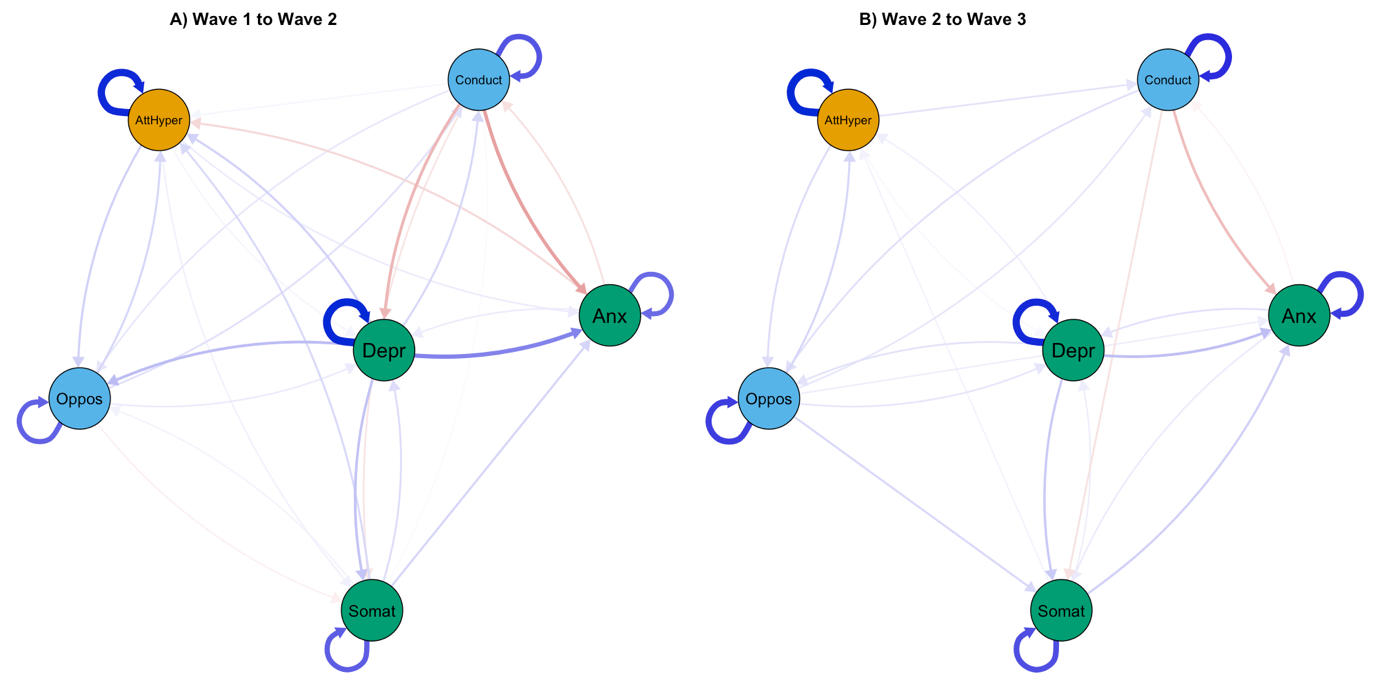
**

*Note.* GenAnx = General Anxiety, OCD = Obsessive-compulsive Disorder, Panic = Panic Disorder, SepAnx = Separation Anxiety, SocPho = Social Phobia, AttHyper = Attention Deficit Hyperactivity Problems, Depr = Depressive Problems, Conduct = Conduct Problems, Oppos = Oppositional Defiant Problems, Somat = Somatic Problems, CLPN = Cross-Lagged Panel Network Analysis.

# Section 6: Executive functioning task regression models

**Figure S10**

*Correlations between all EF measures*

***
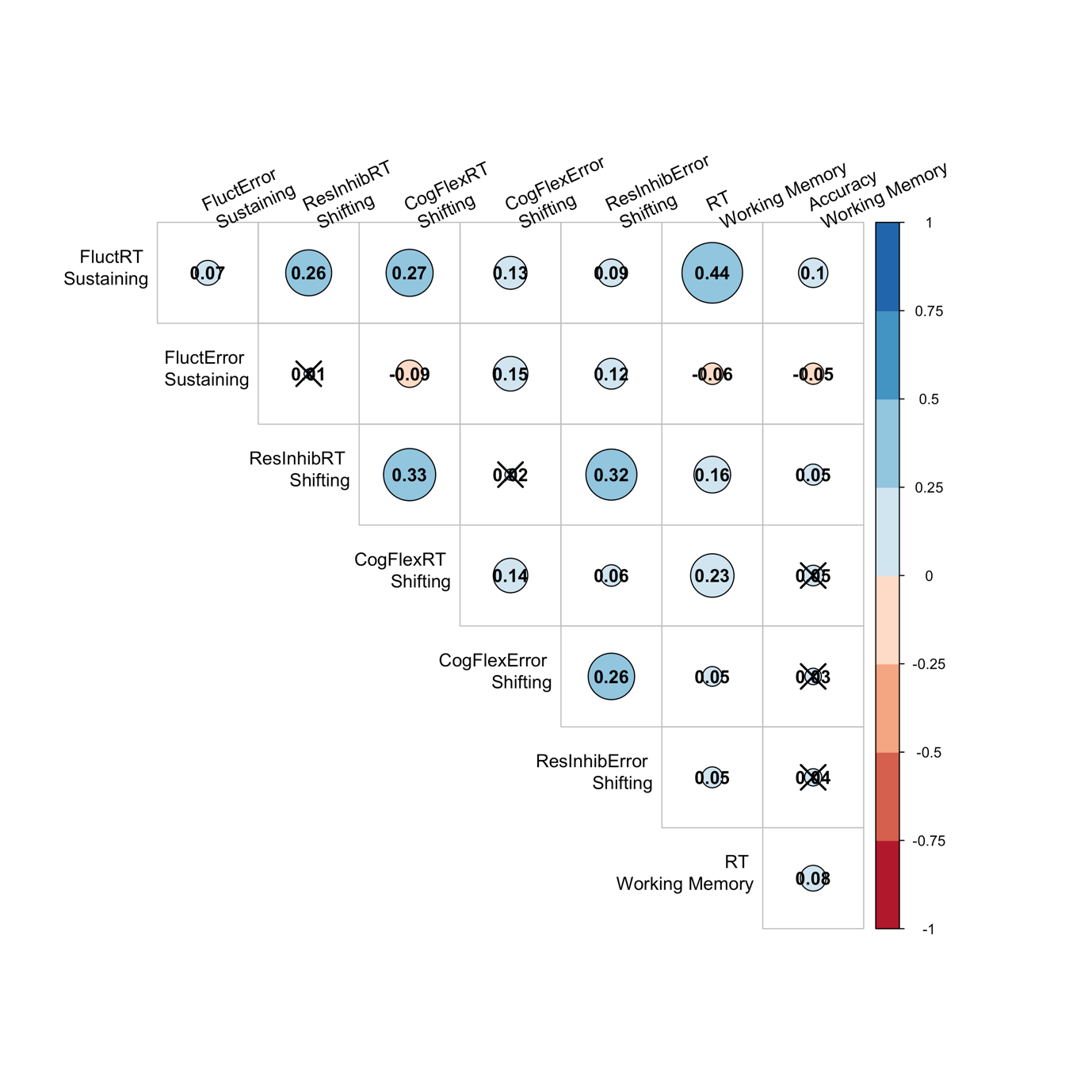
****Note.* Non-significant correlations (*p < 0.05)* are crossed out. RT = Reaction Times, ResInhib = Response inhibition, CogFlex = Cognitive flexibility, Fluct = Fluctuation in tempo, EF = Executive Functioning.

**Table S3**

*Regression Results (Step 1) for Internalizing Symptoms at Wave 2*

| Predictor | Estimate | SE | *z* | *p* |
| --- | --- | --- | --- | --- |
| Sex | -0,43 | 0,04 | -10,9 | <0.01 |
| Internalizing symptoms at wave 1 | 0,49 | 0,02 | 21,31 | <0.01 |
| Externalizing symptoms at wave 1 | -0,01 | 0,02 | -0,58 | 0,562 |

*Note. SE* = Standard Error, Total R^2^ = 0.3.

**Table S4**

*Regression Results (Step 2) for Internalizing Symptoms at Wave 2*

| Predictor | Estimate | SE | *z* | *p* |
| --- | --- | --- | --- | --- |
| Sex | -0,44 | 0,04 | -10,67 | <0.01 |
| Internalizing symptoms at wave 1 | 0,48 | 0,02 | 20,84 | <0.01 |
| Externalizing symptoms at wave 1 | -0,02 | 0,02 | -0,76 | 0,447 |
| Fluctuation in tempo (sustaining) | 0,05 | 0,02 | 1,99 | 0,046 |
| Errors (sustaining) | <0.001 | 0,02 | 0,23 | 0,822 |
| Response inhibition RT (shifting) | 0,01 | 0,02 | 0,57 | 0,569 |
| Cognitive flexibility RT (shifting) | <0.001 | 0,02 | 0,04 | 0,965 |
| Cognitive flexibility errors (shifting) | -0,02 | 0,02 | -0,94 | 0,347 |
| Response inhibition errors (shifting) | -0,01 | 0,02 | -0,37 | 0,711 |
| RT working memory | -0,01 | 0,02 | -0,6 | 0,548 |
| Accuracy/errors working memory | -0,01 | 0,02 | -0,44 | 0,662 |

*Note. SE* = Standard Error, Total R^2^ = 0.3.

**Table S5**

*Regression Results (Step 1) for Internalizing Symptoms at Wave 3*

| Predictor | Estimate | SE | *z* | *p* |
| --- | --- | --- | --- | --- |
| Sex | -0,38 | 0,04 | -9,16 | <0.01 |
| Internalizing symptoms at wave 2 | 0,52 | 0,02 | 22,44 | <0.01 |
| Externalizing symptoms at wave 2 | 0,05 | 0,02 | 1,97 | 0,049 |

*Note. SE* = Standard Error, Total R^2^ = 0.38.

**Table S6**

*Regression Results (Step 2) for Internalizing Symptoms at Wave 3*

| Predictor | | Estimate | SE | *z* | *p* |
| --- | --- | --- | --- | --- | --- |
| Sex | -0,38 | | 0,04 | -8,67 | <0.01 |
| Internalizing symptoms at wave 2 | 0,53 | | 0,02 | 22,53 | <0.01 |
| Externalizing symptoms at wave 2 | 0,05 | | 0,02 | 1,91 | 0,056 |
| Fluctuation in tempo (sustaining) | 0,03 | | 0,03 | 1,18 | 0,237 |
| Errors (sustaining) | -0,04 | | 0,02 | -1,72 | 0,086 |
| Response inhibition RT (shifting) | 0,06 | | 0,03 | 2,56 | 0,01 |
| Cognitive flexibility RT (shifting) | -0,01 | | 0,02 | -0,48 | 0,629 |
| Cognitive flexibility errors (shifting) | 0,04 | | 0,03 | 1,66 | 0,097 |
| Response inhibition errors (shifting) | -0,01 | | 0,02 | -0,22 | 0,827 |
| RT working memory | -0,02 | | 0,02 | -0,76 | 0,448 |
| Accuracy/errors working memory | 0,01 | | 0,02 | 0,41 | 0,683 |

*Note. RT* = Reaction Times, *SE* = Standard Error, Total R^2^ = 0.39.

**Table S7**

*Regression Results (Step 1) for Externalizing Symptoms at Wave 2*

| Predictor | Estimate | SE | *z* | *p* |
| --- | --- | --- | --- | --- |
| Sex | -0,02 | 0,04 | -0,51 | 0,611 |
| Internalizing symptoms at wave 1 | 0,06 | 0,02 | 2,44 | 0,015 |
| Externalizing symptoms at wave 1 | 0,43 | 0,02 | 17,7 | <0.01 |

*Note. SE* = Standard Error, Total R^2^ = 0.22.

**Table S8**

*Regression Results (Step 2) for Externalizing Symptoms at Wave 2*

| Predictor | Estimate | SE | *z* | *p* |
| --- | --- | --- | --- | --- |
| Sex | -0,03 | 0,04 | -0,71 | 0,48 |
| Internalizing symptoms at wave 1 | 0,05 | 0,02 | 2,21 | 0,027 |
| Externalizing symptoms at wave 1 | 0,42 | 0,02 | 16,9 | <0.01 |
| Fluctuation in tempo (sustaining) | 0,05 | 0,03 | 2,02 | 0,043 |
| Errors (sustaining) | 0,04 | 0,02 | 1,89 | 0,059 |
| Response inhibition RT (shifting) | 0,03 | 0,02 | 1,28 | 0,202 |
| Cognitive flexibility RT (shifting) | -0,02 | 0,02 | -1,02 | 0,309 |
| Cognitive flexibility errors (shifting) | 0,01 | 0,02 | 0,29 | 0,77 |
| Response inhibition errors (shifting) | -0,02 | 0,02 | -0,83 | 0,409 |
| RT working memory | -0,01 | 0,02 | -0,57 | 0,569 |
| Accuracy/errors working memory | 0,03 | 0,02 | 1,25 | 0,211 |

*Note. RT* = Reaction Times, *SE* = Standard Error, Total R^2^ = 0.21.

**Table S9**

*Regression Results (Step 1) for Externalizing Symptoms at Wave 3*

| Predictor | Estimate | SE | *z* | *p* |
| --- | --- | --- | --- | --- |
| Sex | 0,09 | 0,04 | 2,13 | 0,034 |
| Internalizing symptoms at wave 2 | 0,01 | 0,02 | 0,48 | 0,634 |
| Externalizing symptoms at wave 2 | 0,57 | 0,02 | 23,69 | <0.01 |

*Note. SE* = Standard Error, Total R^2^ = 0.33.

**Table S10**

*Regression Results (Step 2) for Externalizing Symptoms at Wave 3*

| Predictor | Estimate | SE | *z* | *p* |
| --- | --- | --- | --- | --- |
| Sex | 0,09 | 0,05 | 1,98 | 0,047 |
| Internalizing symptoms at wave 2 | 0,01 | 0,02 | 0,24 | 0,812 |
| Externalizing symptoms at wave 2 | 0,58 | 0,02 | 23,47 | <0.01 |
| Fluctuation in tempo (sustaining) | 0,05 | 0,03 | 1,82 | 0,068 |
| Errors (sustaining) | 0,01 | 0,02 | 0,54 | 0,586 |
| Response inhibition RT (shifting) | 0,02 | 0,03 | 0,91 | 0,361 |
| Cognitive flexibility RT (shifting) | 0,01 | 0,03 | 0,24 | 0,811 |
| Cognitive flexibility errors (shifting) | <0.001 | 0,03 | -0,01 | 0,996 |
| Response inhibition errors (shifting) | 0,01 | 0,03 | 0,38 | 0,702 |
| RT working memory | -0,02 | 0,03 | -0,75 | 0,453 |
| Accuracy/errors working memory | 0,03 | 0,02 | 1,26 | 0,208 |

*Note. RT* = Reaction Times, *SE* = Standard Error, Total R^2^ = 0.34.

# Section 7: Bootstrapping stability analysis

**Figure S11**

*Bootstrapping Results for Cross-Sectional Network at Wave 1*

*
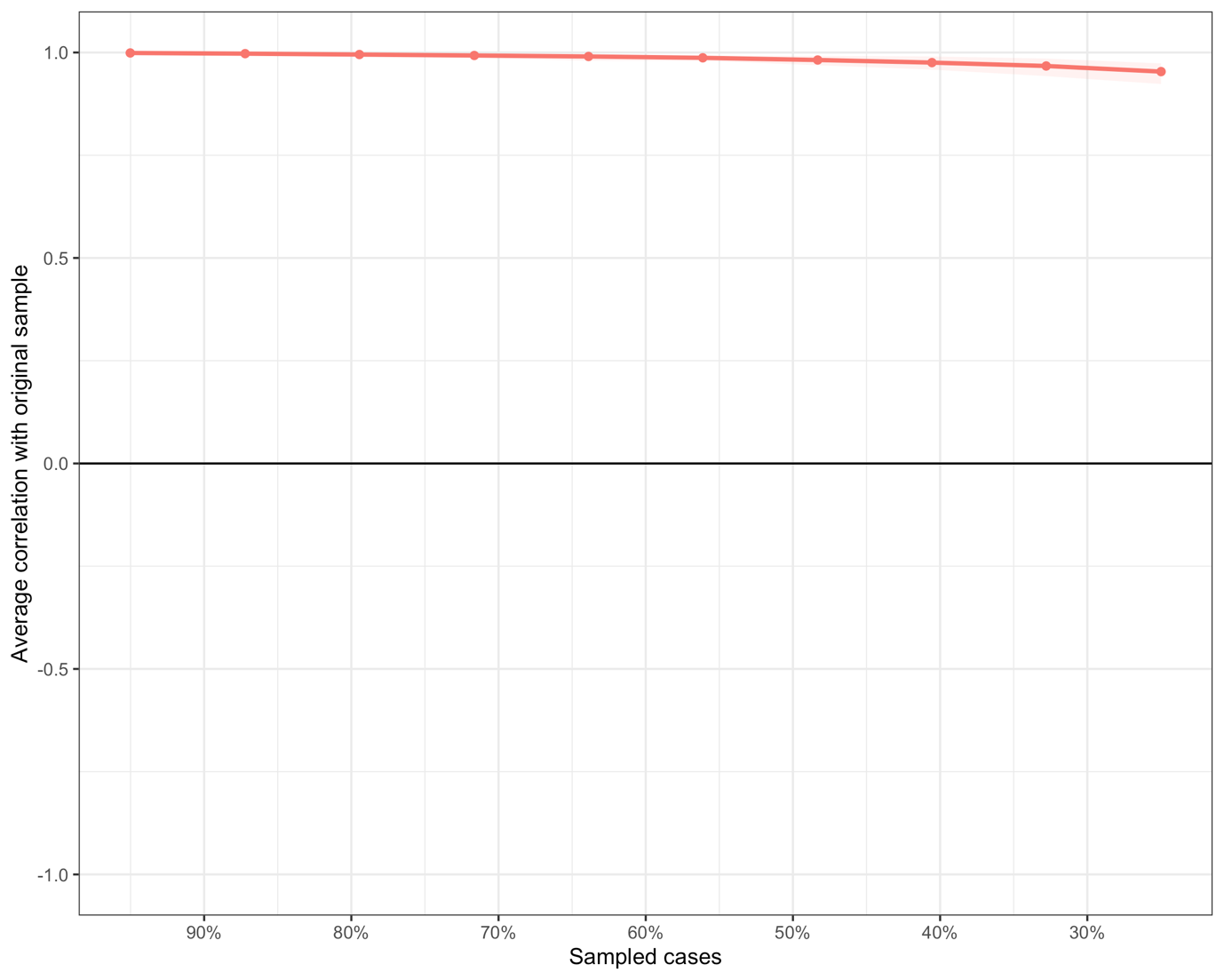
*

**Figure S12**

*Bootstrapping Results for Cross-Sectional Network at Wave 2*

*
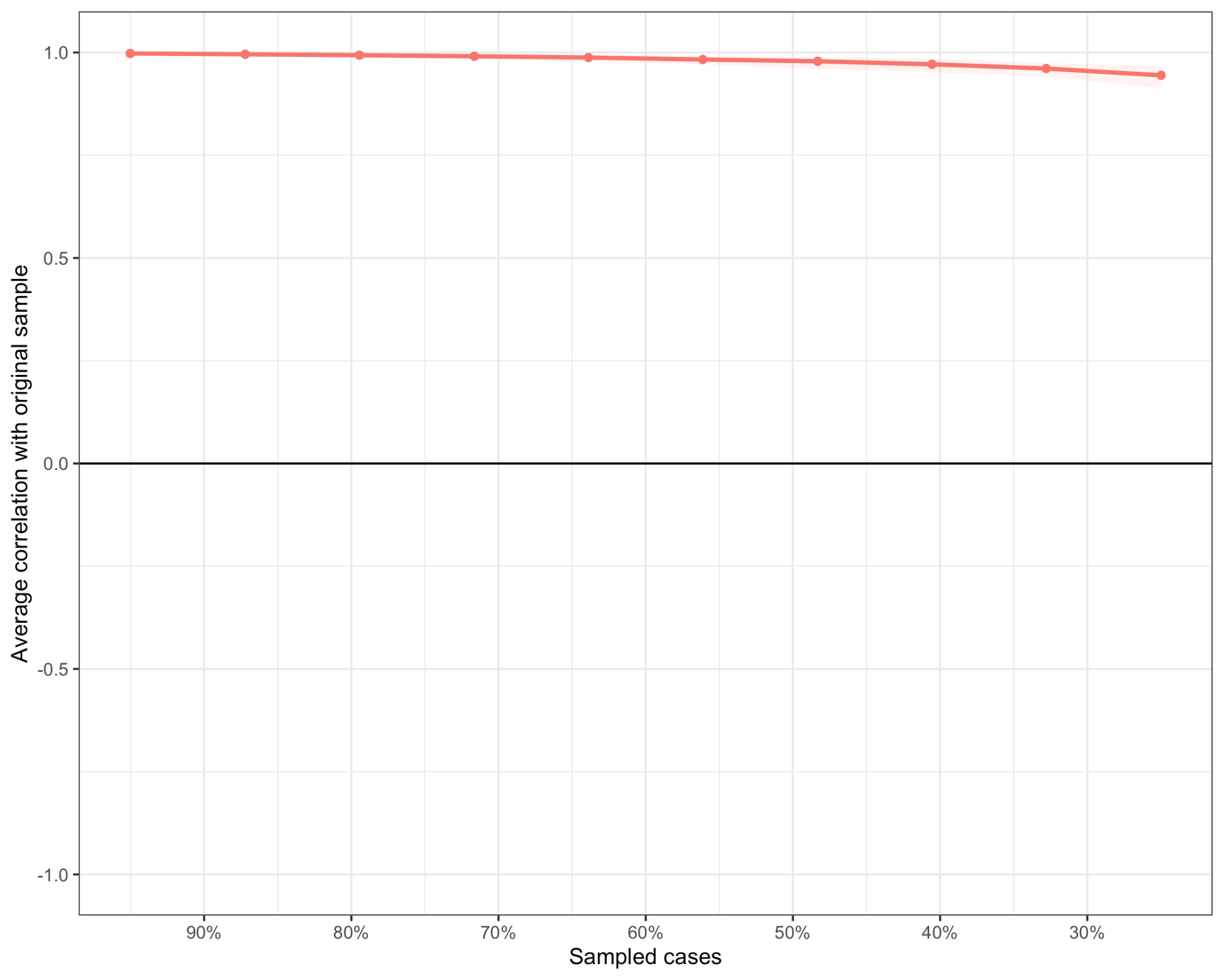
*

**Figure S13**

*Bootstrapping Results for Cross-Sectional Network at Wave 3*

*
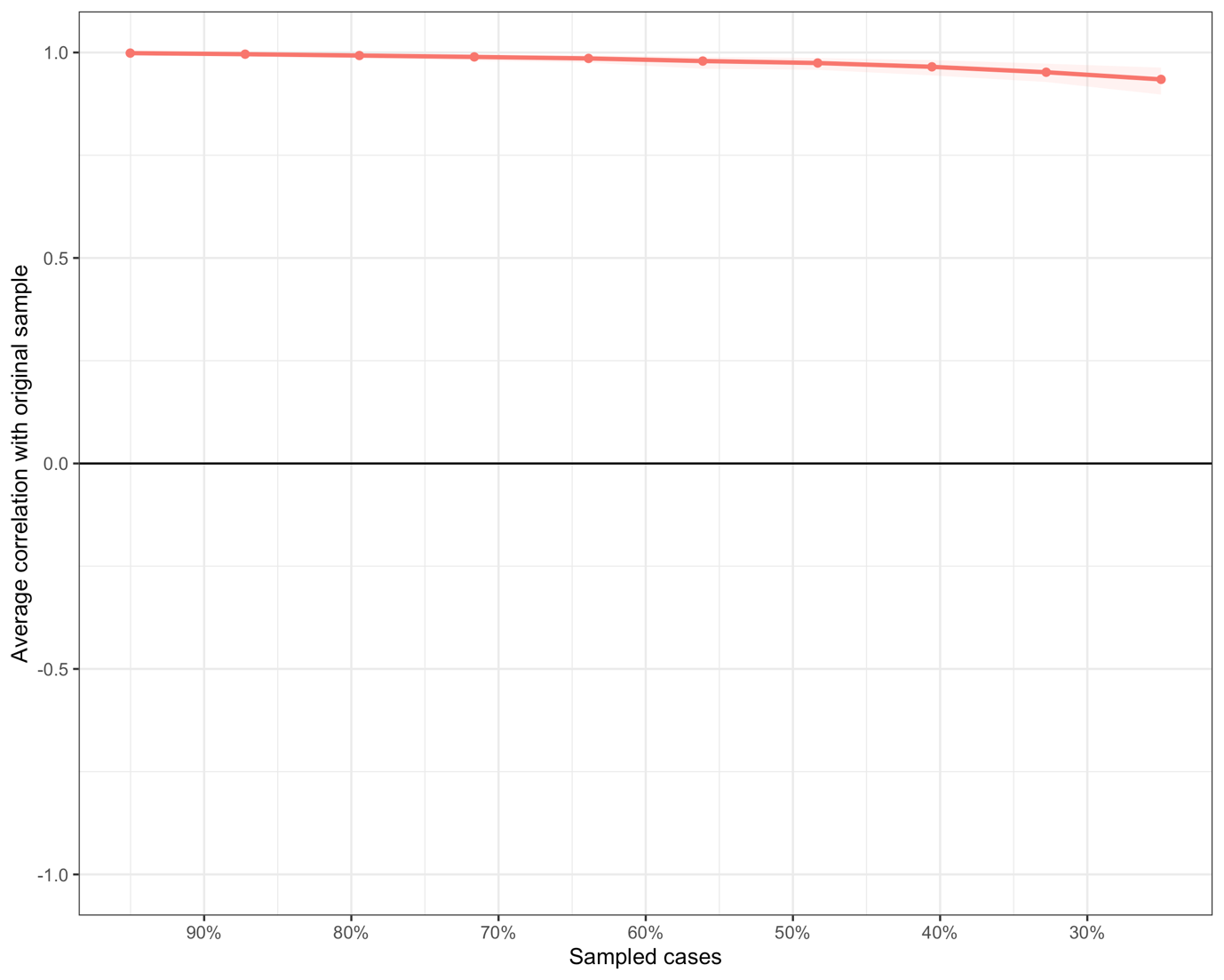
*
